# DNA Protecting Activities of *Nymphaea nouchali* (Burm. f) Flower Extract Attenuate *t*-BHP-Induced Oxidative Stress Cell Death through Nrf2-Mediated Induction of Heme Oxygenase-1 Expression by Activating MAP-Kinases

**DOI:** 10.3390/ijms18102069

**Published:** 2017-09-28

**Authors:** Md Badrul Alam, Mi-Kyoung Ju, Sang-Han Lee

**Affiliations:** 1 Department of Food Science and Biotechnology, Graduate School, Kyungpook National University, Daegu 41566, Korea; mbalam@knu.ac.kr (M.B.A.); jmg8586@naver.com (M.-K.J.); 2 Food and Bio-Industry Research Institute, Kyungpook National University, Daegu 41566, Korea

**Keywords:** antioxidant, *Nymphaea nouchali*, heme oxygenase 1 (HO-1), nuclear factor (erythroid-derived 2)-like 2 (Nrf2), Kelch-like ECH-associated protein 1 (Keap1)

## Abstract

This study was performed to investigate the antioxidant activities of *Nymphaea nouchali* flower (NNF) extract and the underlying mechanism using RAW 264.7 cells. The presence of gallic acid, catechin, epicatechin, epigallocatechin, epicatechin gallate, caffeic acid, quercetin, and apigenin in the NNF was confirmed by high-performance liquid chromatography (HPLC). The extract had a very potent capacity to scavenge numerous free radicals. NNF extract was also able to prevent DNA damage and quench cellular reactive oxygen species (ROS) generation induced by *tert*-Butyl hydroperoxide (*t*-BHP) with no signs of toxicity. The NNF extract was able to augment the expression of both primary and phase II detoxifying enzyme, resulting in combat the oxidative stress. This is accomplished by phosphorylation of mitogen-activated protein kinase (MAP kinase) (p38 kinase and extracellular signal-regulated kinase (ERK)) followed by enhancing the nuclear translocation of the nuclear factor erythroid 2-related factor 2 (Nrf2). This attenuates cellular ROS generation and confers protection from cell death. Altogether, the results of current study revealed that *Nymphaea nouchali* flower could be a source of natural phytochemicals that could lead to the development of new therapeutic agents for preventing oxidative stress associated diseases and attenuating disease progression.

## 1. Introduction

Aerobic organisms use oxygen for their survival; however, in normal physiological and metabolic processes, approximately 5% of oxygen becomes univalently reduced to extremely reactive oxygen-derived free radicals, reactive oxygen species (ROS), and reactive nitrogen species (RNS). These substances are potentially damaging transient chemical species that cause an imbalance in the natural defense systems (both enzymatic and non-enzymatic) by generating oxidative stress, which in turn can lead to a number of physiological disorders, such as Alzheimer’s disease, Parkinson’s disease, inflammation, diabetes, cancer, autoimmune diseases, and digestive system disorders (e.g., gastrointestinal inflammation, ulcers) [1]. Phytochemicals that possess intrinsic antioxidant properties or that directly or indirectly trigger the intracellular signaling pathways may have therapeutic applications for diseases related to oxidative damage [2].

The activation of nuclear factor (erythroid-derived 2)-like 2 (Nrf2) is solely associated with the upregulation of various detoxifying and antioxidant enzymes, such as heme oxygenase-1 (HO-1), NAD(P)H, quinone oxidoreductase (NQO-1), glutathione *S*-transferase (GST), and γ-glutamyl cysteine synthetase catalytic subunit (GCLc) [3,4]. Under resting conditions, Nrf2 is inactive in the cytoplasm and sequestered by Kelch-like ECH-associated protein 1 (Keap1), which hinders the nuclear translocation of Nrf2. On interaction with various inducers, Nrf2 parts from Keap1 and translocates into the nucleus where its bind to antioxidant-related elements (ARE) in the antioxidant and cytoprotective genes promoter region [4]. Additionally, the nuclear translocation of Nrf2 often required the activation of several signaling cascades, such as mitogen-activated protein kinase (MAPK), phosphatidylinositol 3-kinase (PI3K/Akt), and protein kinase C (PKC) [5].

*Nymphaea nouchali* (Burm. f), locally known as “Shapla” in Bangladesh and “Suryeon” in Korea, is an aquatic plant that grows abundantly in mixed populations in almost all shallow natural bodies of water, and has been designated as the national flower of Bangladesh. The whole plant is used for the treatment of liver disorders in Ayurveda. The plant’s leaves, roots, and flowers are used to treat diabetes, blood disorders, heart troubles, and dysentery, and are used as cardiotonics, diuretics, narcotics, and aphrodisiacs [6,7]. The rhizomes and flowers are used as a remedy for kidney problems [8]. The leaves, flowers, and seeds have been found to have antioxidant, antidiabetics, antimicrobial, and hemolytic properties [9] and a novel Ca^+2^-dependent lectin with antiproliferative activity was isolated from the tuber of *N. nouchali* [10]. Astragalin, corilagin, catechin, epicatechin, gallic acid, isokaempferide, kaempferol, quercetin-3-methyl ether, and quercetin have been identified in the flowers [11]. However, no investigations to date have been lead to identify the subset of antioxidant enzymes that are transcriptionally controlled by the *Nymphaea nouchali* flower (NNF), and the mechanisms by which NNF regulates the expression of antioxidant enzymes have yet to be understood. Therefore, we designed the present study to decorticate the potential protective effect of NNF against *tert*-Butyl hydroperoxide (*t*-BHP)-induced oxidative stress in RAW264.7 cells and to characterize the underlying mechanism of HO-1 regulation and induction by NNF via Nrf2 activation and translocation.

## 2. Results

### 2.1. High-Performance Liquid Chromatography (HPLC) Analysis of Nymphaea nouchali Flower (NNF) Extract

**Figure 1:**
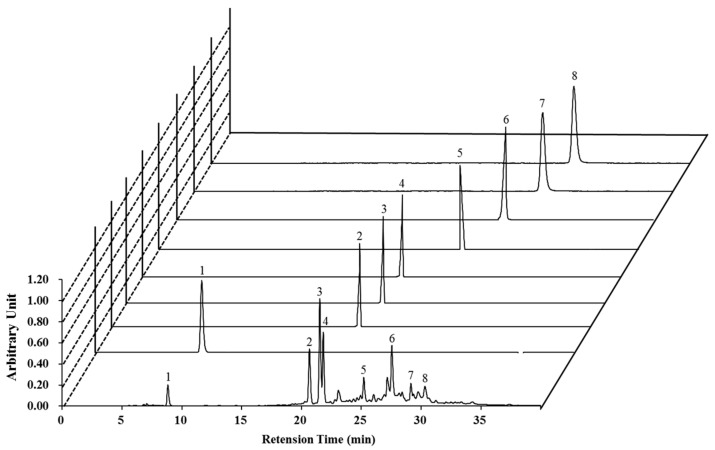
Representative high performance liquid chromatography profile of the methanolic extract of *Nymphaea nouchali* flower with standards monitored at 280 nm. Gallic acid (**Peak 1**), catechin (**Peak 2**), epicatechin gallate (**Peak 3**), epigallocatechin (**Peak 4**), epicatechin (**Peak 5**), caffeic acid (**Peak 6**), quercetin (**Peak 7**), and apigenin (**Peak 8**) are presented.

The high-performance liquid chromatography (HPLC) analysis was carried out using various standard polyphenolics to detect the major polyphenolics in the NNF extract. Interestingly, The LC-UV chromatogram (Figure 1) with diode array detection (DAD) shows that the NNF extract displayed peaks with the same retention times as the following standard polyphenolics: gallic acid (8.27 min), catechin (21.05 min), epigallocatechin (22.15 min), epicatechin gallate (22.30 min), epicatechin (25.83 min), caffeic acid (27.05 min), quercetin (29.50 min), and apigenin (30.13 min). By using the peak areas of known concentrations of standards, the amounts of these polyphenolic compounds in NNF were calculated. As shown in Figure 1, the contents of Gallic acid (2.565%), catechin (7.946%), epicatechin gallate (12.578%), epigallocatechin (8.226%), epicatechin (4.081%), caffeic acid (9.414%), quercetin (3.271%), and apigenin (4.319%). Based on these results, a 10 μg/mL NNF solution contains approximately Gallic acid (1~2 μM), catechin (2~3 μM), epicatechin gallate (3~4 μM), epigallocatechin (1~2 μM), epicatechin (1~2 μM), caffeic acid (4~5 μM), quercetin (0.5~1 μM), and apigenin (1~2 μM).

### 2.2. Radical Scavenging Activities of NNF Extracts

**Figure 2:**
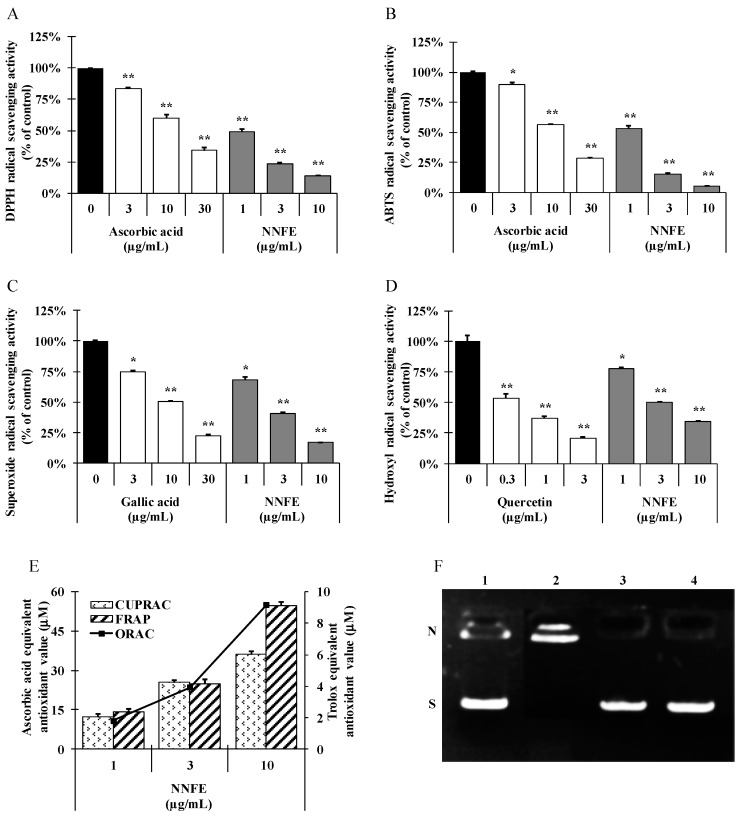
Radical scavenging and DNA protective effects of *N. nouchali* flower (ethylacetate) (NNFE) extract. 2,2-diphenyl-1-picrylhydrazyl (DPPH) radical scavenging (**A**), 2,2′-azino-bis(3-ethylbenzothiazoline-6-sulphonic acid) (ABTS)^•+^ radical scavenging (**B**), superoxide (O_2_^•−^) radical scavenging (**C**), and hydroxyl (OH) radical scavenging (**D**) assays were conducted with NNFE (1, 3, and 10 μg/mL), and ascorbic acid, gallic acid, and quercetin were tested as standard antioxidant compounds. (**E**) Cupric reducing antioxidant capacity (CUPRAC), ferric reducing antioxidant power (FRAP), and oxygen radical absorbance capacity (ORAC) assays were conducted to test the dose-dependent (1, 3, and 10 μg/mL) effect of NNFE. Ascorbic acid equivalent antioxidant capacity was measured for the CUPRAC and FRAP assays, whereas the ORAC activities of the samples were calculated by subtracting the area under the blank curve from the area under the sample curve to obtain the net area under the curve (Net AUC). The trolox equivalent antioxidant capacity was measured as a control. Values are expressed as the mean ± SD (*n* = 3). * *p* < 0.05 and ** *p* < 0.01. Statistical analysis was carried out using student’s *t*-test. (**F**) Agarose gel electrophoretic separation of damaged DNA and the protective effect of NNFE. **1**: DNA alone; **2**: DNA plus Cu (II)-ascorbic acid; **3**: DNA plus Cu (II)-ascorbic acid and NNFE; **4**: DNA plus Cu (II)-ascorbic acid and quercetin; **S**: supercoiled DNA strands; and **N**: nicked DNA strands. Bars: black: Negative control, white: positive control, gray: sample.

Depending on the nature of the mechanism, antioxidants directly and/or indirectly prevent the oxidative stress-induced cellular damage. Direct antioxidant activity involves the donation of hydrogen or electrons to quench free radical, reactive oxygen, and nitrogen species. In contrast, indirect antioxidant activity protects oxidative stress-induced cellular damage by upregulation of phase II detoxifying and antioxidant genes [2]. To investigate the direct antioxidant potential of *N. nouchali* flower extracts, we tested the scavenging activity of various radicals such as 2,2-diphenyl-1-picrylhydrazyl (DPPH^•^), 2,2′-azino-bis(3-ethylbenzothiazoline-6-sulphonic acid) (ABTS)^•+^, superoxide, and hydroxyl radicals. Methanolic extract of *N. nouchali* flowers and its organic soluble fractions significantly scavenged DPPH^•^, a stable organic nitrogen radical, in a dose-dependent manner (Figure 2A and supplementary data Figure S2). Furthermore, after performing a mixed electron transfer and hydrogen atom transfer assay that reduced the radical cation from ABTS^•+^, we found that all extracts of NNF dose-dependently reduced the occurrence of ABTS^•+^ (Figure 2B and supplementary data Figure S2). Phenazine methosulfate (PMS)-NADH superoxide generating system was carried to assess the superoxide radical-scavenging ability of *N. nouchali* flower extracts (Figure 2C and supplementary data Figure S3). Hydroxyl-radical is the most reactive oxygen centered species and causes severe damage to adjacent biomolecule. Hydroxyl radical scavenging activity was estimated by generating the hydroxyl radicals using Fenton reaction and Figure 2D (supplementary data Figure S3) demonstrated that NNF dose dependently inhibited the production of hydroxyl radicals. Additionally, NNF extracts can donate electrons; therefore, we assessed the Cupric reducing antioxidant capacity (CUPRAC), ferric reducing antioxidant power (FRAP), and oxygen radical absorbance capacity (ORAC) of the sample and found that *N. nouchali* flower extracts has a strong reducing power capacity in a concentration-dependent manner (Figure 2E and supplementary data Figures S4 and S5). Based on these observations, we postulate that NNF extracts has a very potent capacity to donate electrons or transfer hydrogen atoms in order to scavenge various free radicals. Furthermore, we also tested the radical scavenging activities of the identified constituents of NNF, at their putative concentrations in NNF. All identified compounds showed a strong radical scavenging activity in order of caffeic acid ≥ NNFE > epigallocatechin > epicatechin > gallic acid ≥ catechin > epicatechin gallate > quercetin ≥ apigenin (supplementary data Figure S6).

### 2.3. DNA-Protective Activity of N. nouchali Flower (Ethylacetate) Extract (NNFE)

Oxidative stress perpetually attacks the cells and DNA of the human body, and is the primary cause of damage induced by ROS and RNS. ROS interact with biological molecules and inhibit the key antioxidant and DNA repair enzymes resulting in disruption of the normal synthesis and repair of DNA [12]. To test whether NNFE has DNA protecting activity, NNFE extract was assessed for their ability to prevent oxidative damage against λ DNA. Hydroxyl radicals generated by Fenton’s reaction are known to cause DNA damage, as demonstrated by the absence of a distinct DNA band in Figure 2F lane 2, where only a smear of degraded DNA is observed. NNFE effectively mitigated oxidative stress and protected the DNA from hydroxyl radicals generated by Fenton’s reactions, as confirmed by the presence of DNA bands in Figure 2F lane 3, suggesting that NNFE strongly prevented DNA damage induced by various oxidative stresses.

### 2.4. NNFE Augments Cell Viability and Attenuates Cellular Oxidative Stress Induced by tert-Butyl Hydroperoxide (t-BHP)

**Figure 3:**
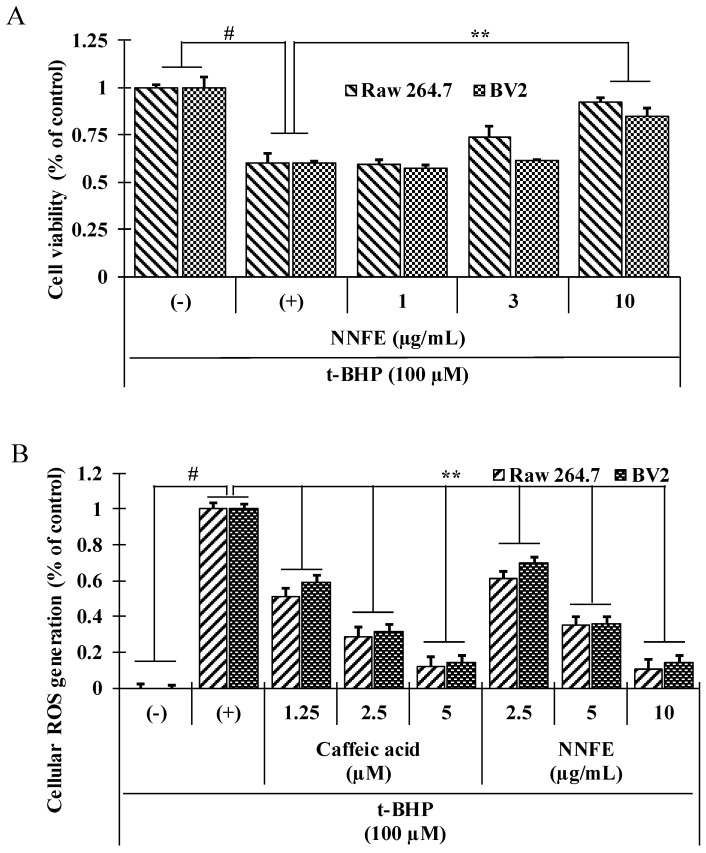
Protective effects of NNFE against *tert*-Butyl hydroperoxide (*t*-BHP) induces cell viability and intracellular reactive oxygen species (ROS) generation. Cells were treated with NNFE (1–10 μg/mL) for 12 h and then exposed to 100 μM *t*-BHP for 12 h, and cell viability percentage was determined by 3-(4,5-dimethylthiazol-2-yl)-2,5-diphenyltetrazolium bromide (MTT) assay for RAW 264.7 cells and BV2 cells (**A**). Cells were treated for 12 h with the indicated concentrations of NNFE and caffeic acid before treatment with 100 μM *t*-BHP. Intracellular ROS was measured by monitoring the dichlorodihydrofluorescein (DCF) fluorescence intensity for RAW 264.7 cells and BV2 cells (**B**). Values are expressed as the mean ± SD (*n* = 3). # *p* < 0.001 compared to control, ** *p* < 0.05 compared to *t*-BHP treatment. Statistical analysis was carried out using student’s *t*-test. (−): no treatment (+): treatment with *t*-BHP.

As NNFE has the highest radical scavenging activity and DNA protecting activity among all extracts, we next tested the cellular viability and the ability of NNFE to scavenge *t*-BHP-induced cellular ROS. *t*-BHP, a prooxidant compound, is generally utilized as a substance for assessment of mechanisms of oxidative stress-induced cell damages in cells and tissues [13]. Cell viability of over 95% was observed at NNFE concentrations up to 50 µg/mL (Supplementary data Figure S7). NNFE treatment (1, 3, and 10 μg/mL) significantly increased cell viability by inhibiting *t*-BHP-induced cell death of RAW 264.7 cells and BV2 cells in a dose-dependent manner (Figure 3A). In addition, we tested the effects of all identified constituents, at their putative concentration in NNFE, on *t*-BHP-induced cellular ROS production (Supplementary data Figure S9) indicating that NNFE showed the cellular ROS scavenging effects due to the presence of caffeic acid. In addition, as shown in Figure 3B, treatment with NNFE significantly attenuated the ROS generation in a dose-dependent manner comparable with the similar effects of caffeic acid (5 μM).

### 2.5. Effects of NNFE on Antioxidant Enzyme Expression in RAW 264.7 Cells

**Figure 4:**
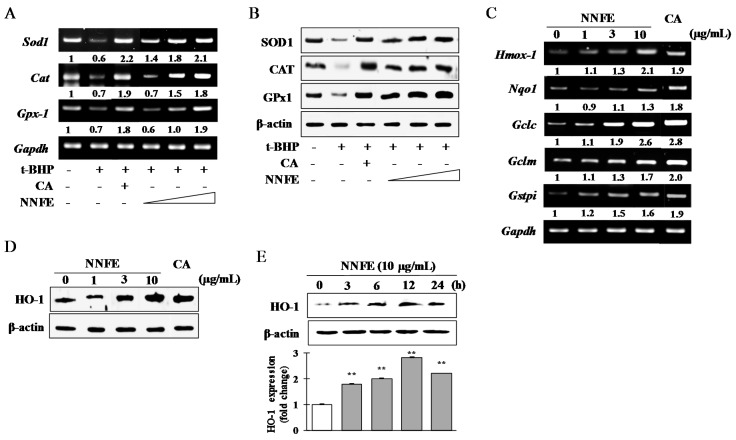
Analysis of primary and phase II antioxidant and detoxifying enzymes. RAW 264.7 cells were pretreated for 12 h with NNFE followed by 12 h treatment with 100 μM *t*-BHP. Primary antioxidant enzyme mRNA expression (**A**) and protein expression (**B**) were analyzed by reverse transcription-polymerase chain reaction (RT-PCR) and western blot, respectively. NNFE (1, 3, and 10 μg/mL) was applied from left to right. RAW 264.7 cells were pretreated for 24 h with NNFE. The mRNA of phase II antioxidant and detoxifying enzymes (**C**) were measured by RT-PCR. The effect on heme oxygenase 1 (HO-1) protein levels in a concentration-dependent (**D**) and time-dependent (**E**) manner was analyzed by western blot. The symbol “**” indicates statistical significance compared to control as determined by one-way ANOVA (** *p* < 0.01). CA: Caffeic acid 5 μM. SOD1: superoxide dismutase 1; CAT: chloramphenicol acetyl transferase; GPx1: glutathione peroxidase 1. (−): no treatment (+): treatment with *t*-BHP, white triangle: concentration low to high.

To investigate where NNFE had the capacity to upregulate the expression of primary antioxidant enzymes such as superoxide dismutase 1 (SOD1), chloramphenicol acetyl transferase (CAT), and glutathione peroxidase 1 (GPx-1), RAW 264.7 cells were pretreated with NNFE (1, 3, and 10 µg/mL) for 2 h and subsequently co-incubated with *t*-BHP for an additional 22 h. As described in Figure 4A, reverse transcription-polymerase chain reaction (RT-PCR) analysis revealed that *t*-BHP treatment strongly mitigated the expression of mRNA of *SOD1*, *CAT* and *GPx-1* whereas NNFE treatment dose-dependently regenerated of these mRNA and as expected the upregulated protein levels of SOD1, CAT, and GPx-1, was confirmed by western blot analysis (Figure 4B). Furthermore, NNFE treatment enhanced the mRNA expression of the phase II detoxifying enzyme-encoding genes *Hmox-1*, *Nqo1*, *GCLc*, *GCLm*, and *Gstpi* (Figure 4C) and also western blot analysis confirmed the augmented protein levels of HO-1 (Figure 4D). In addition, time-dependent western blot analysis revealed that HO-1 expression significantly increased from 3 to 24 h, and peaked at 12 h after NNFE treatment. (Figure 4E; quantifications and statistical analyses are presented in adjacent graphs). These data suggest that NNFE might be showed its antioxidant activity through augmentation of the expression of both mRNA and protein levels of primary antioxidant enzymes and phase II detoxifying enzymes.

### 2.6. Effects of NNFE on Phase II Enzymes Mediated by Nuclear Factor (Erythroid-Derived 2)-Like 2 (Nrf2) Nuclear Translocation in RAW 264.7 Cells

**Figure 5:**
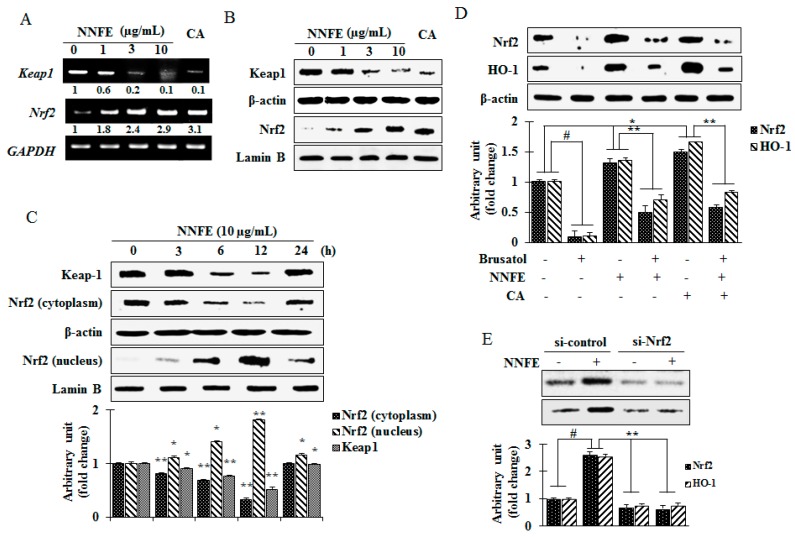
Effect of NNFE on nuclear translocation of nuclear factor erythroid 2-related factor 2 (Nrf2)-mediated HO-1 expression. RAW 264.7 cells were pretreated for 24 h with NNFE. The mRNA expression (**A**) and protein expression (**B**) of Nrf2 and Kelch-like ECH-associated protein 1 (Keap1) were measured by RT-PCR and western blot, respectively. (**C**) The time-dependent effect on the protein levels of cytosolic and nuclear Nrf2 was analyzed by western blot. (**D**) Cells were treated with an Nrf2 inhibitor (brusatol) with and without NNFE, and Nrf2 and HO-1 protein levels were analyzed by western blot. (**E**) Cells were treated with a siRNA of Nrf2 (si-Nrf2) with and without NNFE, and Nrf2 and HO-1 protein levels were analyzed by western blot. Statistical values are expressed as the mean ± SD (*n* = 3). # *p* < 0.001, and * *p* < 0.05 compared to no treatment, ** *p* < 0.01 compared to brusatol treatment. Statistical analysis was carried out using one-way ANOVA. CA: Caffeic acid 5 μM. (−): no treatment (+): treatment with inhibitor and/or sample.

Noteworthy that ARE is found in the promoters of genes that encode phase II detoxifying and antioxidant enzymes such as HO-1. Therefore, genes regulated by the ARE encode proteins that help control of the cellular redox status and protect the cells from oxidative damage [3]. Nrf2 is an important transcription factor that regulates ARE-driven HO-1 gene expression [4]. Under resting condition, Nrf2 is generally fastened in the cytoplasm by the Keap1 protein. In response to inducer, Nrf2 is set free and translocates to the nucleus to upregulate the phase II detoxifying enzymes [5]. In present study, NNFE treatment increased the mRNA level of Nrf2, whereas Keap1 mRNA levels were mitigated in a dose-dependent manner (Figure 5A). In addition, NNFE treatment significantly downregulated Keap1 protein expression in a dose-dependent (Figure 5B) and time-dependent (Figure 5C) manner. However, NNFE treatment was markedly augmented the nuclear Nrf2 protein expression in association with decreased cyto-Nrf2 levels (Figure 5B). Furthermore, a time course study revealed that NNFE caused time-dependent increases in Nrf2 protein levels, peaking at 12 h after NNFE treatment (Figure 5C). To confirm that NNFE activates phase II enzymes through Nrf2, cells were treated with brusatol (a specific Nrf2 inhibitor) and/or transfected with small interfering RNA (siRNA) of Nrf2 (si-Nrf2) before NNFE treatment. As expected, both brusatol and si-Nrf2 significantly inhibited Nrf2 protein levels (Figure 5E), and the addition of NNFE did not exhibit any further effects (Figure 5D,E). Moreover, HO-1 induction by NNFE was also efficiently abolished in both brusatol and si-Nrf2 treated cells (Figure 5D,E). These results revealed that NNFE treatment disrupts the Nrf2-Keap1 complex in cytoplasm, freeing Nrf2 to undergo nuclear translocation and upregulate HO-1 expression.

### 2.7. NNFE Activates Nrf2 via Phosphorylation of Mitogen-Activated Protein Kinase (MAPK) to Attenuate Oxidative Stress

**Figure 6:**
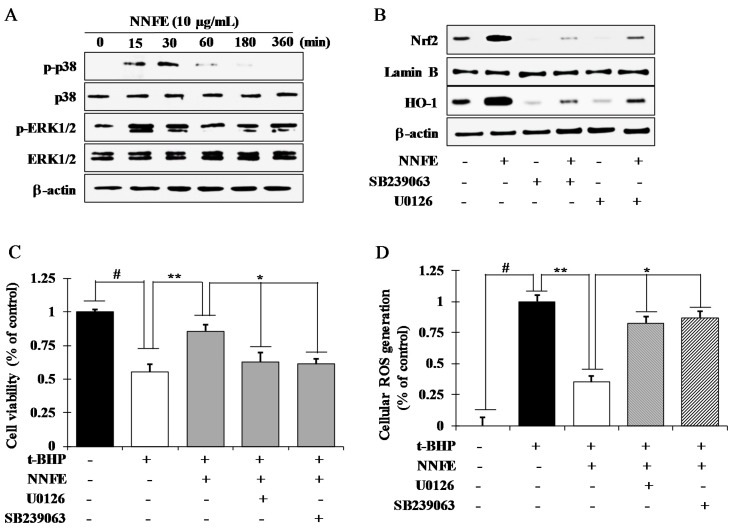
NNFE extract activates the translocation of Nrf2 by activating extracellular signal-regulated kinase 1 and 2 (ERK1/2) and p38. (**A**) RAW 264.7 cells were pretreated with 10 μg/mL NNFE for the indicated time and kinase activation was analyzed by western blot. (**B**) Cells were treated with 10 μg/mL NNFE in the presence and absence of specific inhibitor U0126 and SB239063 and the protein level of Nrf2 and HO-1 were analyzed by western blot. Following co-treatment with NNFE, U0126, and SB239063 for 12 h, cells were challenged with *t*-BHP for an additional 12 h, and then cell viability (**C**) and intracellular ROS generation (**D**) were analyzed. Statistical values are expressed as the mean ± SD (*n* = 3). # *p* < 0.001 compared to no treatment, ** *p* < 0.01 compared to *t*-BHP treatment, * *p* < 0.05 compared to NNFE treatment. Statistical analysis was carried out using one-way ANOVA. (−): no treatment, (+): treatment with inhibitor and/or sample.

To elucidate the upstream signaling pathway involved in NNFE-mediated activation of the Nrf2/Keap1 system, cells were treated with NNFE for indicated time interval, and the phosphorylation of mitogen-activated protein kinase (MAP kinase) (extracellular signal-regulated kinase 1 and 2 (ERK1/2), c-Jun N-terminal kinase (JNK) and p38) was assessed by western blot analysis. As shown in Figure 6A, NNFE treatment enhanced ERK1/2 and p38 phosphorylation after 15 and 30 min, respectively. However, phosphorylated JNK was not detected in NNFE-treated cells (data not shown). In addition, to confirm the upstream signaling cascade involved in the induction of Nrf2 activity and HO-1 expression, specific inhibitors (SB239063 for p38 and U0126 for ERK) were applied to cells treated with NNFE. As expected, inhibition of p38 and ERK1/2 pathways strongly mitigated the capacity of NNFE to enhance the nuclear Nrf2 accumulation and protein expression of HO-1 (Figure 6B). Based on these observations, it is inferred that NNFE treatment augmented the Nrf2-mediated expression of HO-1 through the activation of ERK and p38 signaling cascade in RAW 264.7 cells.

Finally, we attempted to determine whether NNFE could attenuate oxidative cell death through the activation of ERK1/2 and p38 signaling. Interestingly, as shown Figure 6C,D, pretreatment with NNFE significantly mitigated cell death and ROS generation compared to *t*-BHP-treated cells. In contrast, this effect was partially blunted in the presence of ERK1/2 and p38 inhibitors, suggesting the involvement of ERK1/2 and p38 signaling in NNFE-mediated activation of Nrf2-induced HO-1 expression as well as in cytoprotection against oxidative stress.

## 3. Discussion

Molecular oxygen (O_2_) is essential to carry out cellular processes in all aerobic organisms and cells can generate partially reduced forms of O_2_ which is referred as ROS, during respiration as well as enzymatic reaction. They act as toxic intracellular species and induce oxidative stress, resulting in an imbalance between free radical production and elimination through cellular defense mechanisms [14]. Mounting evidence suggests that strong chemically reactive ROS function exclusively as cellular destructive agents, generally reacting with lipids, proteins, and DNA [15]. Using in vitro chemical assays, we evaluated the antioxidant effects of NNF extract and the underlying mechanism by which NNF extract mitigates oxidative stress. Oxidative stress was induced by *t*-BHP treatment, which regulates primary and phase II detoxifying enzymes through the Keap1-Nrf2 pathway in RAW 264.7 cells by activating MAPK (ERK1/2 and p38) signaling pathways.

Free radicals are believed to cause DNA double-strand breaks and damage, which eventually contribute to carcinogenesis, mutagenesis, and cytotoxicity. Numerous researchers have reported similar findings, and have used plant extracts and fractions for DNA protection against oxidative damage [12]. Antioxidant activity is generally attributed to the phytochemicals present in the extract, a variety of mechanisms in plants, and the synergy between them. Thus, the antioxidant activity of plant extracts cannot be evaluated by a single method. Therefore, to explore the antioxidant mechanisms of NNF extracts, DPPH, ABTS, O_2_^•−^, and HO radical scavenging assays, and FRAP, CUPRAC, and ORAC assays were carried out. The results demonstrate that NNF extracts have a broad range of antioxidant properties. The hydroxyl radical, which is generated by the Fenton reaction using Fe^3+^-ascorbate-EDTA-H_2_O_2_ system. It is most toxic among all radicals and all classes of biological macromolecules such as lipids, proteins and nucleic acid are nonspecifically oxidized by it, resulting in the development of mutagenesis, carcinogenesis and aging [15]. Plants with higher hydroxyl radical-quenching ability play an important role in health: their consumption can control and mitigate the devastating effects of oxidative stress. The impact of our results is highlighted by our finding that NNFE can prevent DNA damage caused by hydroxyl radicals (Figure 2F). Mounting evidence suggests that phenolic compounds found in both edible and inedible plants have diverse biological effects (e.g., antioxidant activity), mainly due to their redox properties [16]. Likewise, flavonoids, a diverse and widespread group of polyphenolics in nature, and possess a broad spectrum of chemical and biological activities, including antioxidant properties [17]. Therefore, it is worthwhile to determine the total phenolic compound and flavonoid content in the plant chosen for the study (Supplementary data S1). The content of total phenolics and flavonoids in the extracts of NNF was determined by first using the Folin-Ciocalteu assay and aluminum chloride colorimetric method, respectively. Next, regression equations of calibration curve were used: total phenolic content was calculated according to the gallic acid equivalent method (y = 0.0514x + 0.0008; *r*^2^ = 0.9935), and flavonoid content was measured according to the catechin equivalent method (y = 0.012x + 0.0031; *r*^2^ = 0.9984). To calculate the correlation between polyphenols, flavonoids, and antioxidant activity of NNF, the Pearson coefficient (*ρ*) method and linear regression analysis (data not shown) was assessed. Noteworthy stated that a negative *ρ* value (−1) represents the perfect positive correlation between polyphenols and free radical scavenging ability using IC_50_. The results revealed very strong correlations for DPPH, ABTS, and OH scavenging activity (*ρ* = −0.872, −0.871, and −0.935, respectively); and moderate for superoxide scavenging activity (*ρ* = −0.762). These data are in accordance with other studies that show that higher phenol content augments the antioxidant activity [18], and reveal a linear correlation between phenolic content and antioxidant activity [19].

For the evaluation of oxidative damage, cells were exposed to *t*-BHP, a short-chain analogue of lipid peroxide. Decomposition of *t*-BHP in biological systems evokes lipid peroxidation chain reactions, damage to DNA, and depletion of cell glutathione (GSH) content, resulting in cell damage and apoptosis [13]. Numerous scientific reports have revealed that pretreatment of cells with natural phytochemicals prevent oxidative stress-induced cell toxicity [20,21]. Consistent with these reports, pretreatment of both RAW 264.7 and BV2 cells with NNFE significantly attenuated cell death and intracellular ROS generation induced by *t*-BHP (Figure 3; supplementary data Figure S8). Furthermore, mitochondrial electron transport chain reaction can generate the cytosolic superoxide radical (O_2_^−^) from O_2_ through one-electron reduction pathway. It is well known that SOD1 is rapidly converted the O_2_^−^ into H_2_O_2_ which is subsequently detoxified to H_2_O by GPx and CAT [15,22]. Continuous exposure of oxidative stress may cause the damage of these primary antioxidant enzyme, which play a crucial role in cellular homeostasis during cell proliferation, and can develop various degenerative diseases [23]. In this study, NNFE treatment significantly increased both the mRNA and protein level of the antioxidant enzymes SOD1, CAT, and GPx-1 in RAW 264.7 cells and BV2 cells (Figure 4A,B, and Supplementary data Figure S10, respectively), indicating that NNFE has the ability to maintain cellular homeostasis and to protect the cell from oxidative stress. Mounting evidence suggests that polyphenolic compounds or food rich in polyphenolic content increase the activity of SOD1, CAT, and GPx in vitro and in vivo, resulting in the attenuation of oxidative stress [24]. Likewise, flavonoids exhibit antioxidant activity by inhibiting ROS/RNS forming enzymes and by directly scavenging ROS/RNS through the upregulation of antioxidant enzymes [25]. Numerous scientific reports suggest that the flowers of *Nymphaea* sp. are rich in phenol and flavonoid compounds such as galic acid, *p*-coumaric acid, myricetin, various myricetin glycosides, quercetin glycosides, kaempferol, naringenin, luteolin, apigenin, and tannin compounds (e.g., catechin, epigallocatechin, and epicatechin gallate) [26,27]. Based on these findings, we hypothesized that phenolic compounds and flavonoids might be major components of NNF, since polyphenol and flavonoids typically have strong antioxidant properties. To gain insight into the phytochemicals present in NNF, HPLC analysis was employed with standard phenolic and flavonoid compounds. We found that galic acid, catechin, epigallocatechin, epicatechin gallate, epicatechin, caffeic acid, quercetin, and apigenin are present in NNF (Figure 1) and among them caffeic acid in NNFE play a lead role in preventing oxidative stress-induced cell death.

GCLc and GCLm play the pivotal role in catalyzing GSH synthesis. Numerous scientific studies suggested that the decrease in expression of GSH is considered to be a critical biomarker of the progression of various life threatening diseases such as cancer, obesity, diabetes, neurodegenerative disease, and age-related macular degeneration [28]. However, heme is converted to a powerful pro-oxidant biliverdin and subsequently to a strong antioxidant bilirubin by HO-1. Furthermore, HO-1 levels have been found to decrease with increasing oxidative stress [29]. Following the treatment of RAW 264.7 cells with NNFE, a significant increase in phase II enzymes at both the mRNA and protein level was observed (Figure 4C–E, supplementary data Figure S11). Several nutrients with antioxidant properties, such as caffeic acid, catechin, epigallocatechin gallate, epicatechin, quercetin, luteolin, and apigenin, have been reported to induce HO-1 expression and attenuate oxidative stress-induced cell death [30,31]. Therefore, we presumed that the augmentation of phase II enzymes expression might be another mechanism accounting for the benefit of NNFE against oxidative stress. Thus, to explore this mechanics in detail, the mRNA and protein levels of Keap-1 and Nrf2, the key regulators of phase II enzyme activation, were examined. Nrf2 is sequestered by Keap1 in the cytosol and constitutively targeted for poly-ubiquitination under a basal condition. Electrophilic and oxidative stress causes disruption of the Nrf2-Keap1 complex, enabling Nrf2 to translocate to the nucleus and activate phase II detoxifying genes [4]. In this study, inhibition of Nrf2 mitigated the induction of HO-1 by NNFE (Figure 5D,E). Furthermore, NNFE treatment was regulated both Nrf2 and Keap1 protein levels; however, after 12 h, NNFE treatment promoted Nrf2 nuclear translocation, accompanied by a decrease in cyto-Nrf2 concentration (Figure 5C). Moreover, treatment of NNFE caused significant downregulation of Keap1, suggesting that NNFE could activate phase II enzymes by disrupting the Keap1-Nrf2 complex (supplementary data Figure S12). These results suggested that NNFE pretreatment could be modified the cysteine residues of Keap1 resulting in the conformational change of Keap1 and promotes the degradation of Keap1. Therefore, the interaction between NNFE and Nrf2-Keap1 complex may mimic the action of other Nrf2 inducer such as *Gingko biloba* extract. However, details mechanisms of the interaction between NNFE and Keap1 require further investigation. *G. biloba* extract activates Nrf2-mediated phase II enzyme expression in Hepa1c1c7 and HepG2 cells [32]. Polyphenolic compounds such as galic acid, caffeic acid, catechin, and epicatechin are also believed to attenuate oxidative stress-induced liver injury through Nrf2-mediated HO-1 expression [33,34]. Likewise, quercetin, luteolin, and apigenin modulate Nrf2 nuclear translocation and ARE-dependent gene expression of HO-1 in BV2 and PC12 cells [31,35].

It is notably that, some signaling pathways such as PI3K/AKT and MAP kinase pathways are involved to interact with the Keap1/Nrf2/ARE system and help to regulate phase II gene expression [13]. In the current study, phosphorylation of ERK1/2 and p38 by NNFE was observed over time periods ranging from 15 to 360 min. ERK1/2 and p38 inhibitors efficiently blocked the expression of Nrf2 and HO-1 induced by NNFE. Therefore, it is inferred that the Nrf2 activation induced by NNFE is dependent on ERK and p38 activation. Moreover, according to previous reports, dietary antioxidants can activate a number of cellular kinases, such as MAPKs and PI3K/Akt, which are responsible for cell survival against oxidative stress [36]. In the present study, ERK1/2 and p38 inhibitors significantly abolished the protective effects of NNFE on *t*-BHP-induced cell death and ROS generation. Altogether, our results suggest that activation of ERK1/2 and p38 pathways might be involved in the cytoprotective effects of NNFE against oxidative stress.

## 4. Materials and Methods

### 4.1. Plant Materials and Extraction

*N. nouchali* flowers were collected from the lake area of Jessore district in Bangladesh during August 2015 and taxonomically were identified by the National Herbarium of Bangladesh (voucher specimen no. 35,453). It is stored in our laboratory for future reference. The dried and coarsely powdered flowers (100 g) were extracted with methanol under reflux for 3 h (three times) and dried in a rotary vacuum evaporator. The methanol extract residue (NNFM) (15.12 g) was suspended in 1 L of deionized H_2_O and partitioned sequentially with n-hexane, chloroform, and ethyl acetate using funnels in a stepwise manner. After vacuum filtration, the solutions were concentrated in a rotary vacuum evaporator and the n-hexane fraction (NNFH) (2.10 g), chloroform extract (NNFC) (3.54 g), ethyl acetate fraction (NNFE) (6.25 g) and aqueous fraction (NNFW) (3.11 g) were dissolved in deionized H_2_O at a concentration of 30 mg/mL.

### 4.2. Drugs and Chemicals

3-(4,5-dimethylthiazol-2-yl)-2,5-diphenyltetrazolium bromide (MTT), *tert*-Butyl hydroperoxide (*t*-BHP), 2,2-diphenyl-1-picrylhydrazyl (DPPH), phenazine methosulfate (PMS), 2,2′-azino-bis(3-ethylbenzothiazoline-6-sulphonic acid) (ABTS), 2′,7′-Dichlorofluorescin diacetate (DCFH-DA), dimethylsulfoxide (DMSO) and phosphate buffered saline (PBS, pH 7.4) were purchased from Sigma Aldrich (St. Louis, MO, USA). Dulbecco’s modified Eagle’s medium (DMEM), fetal bovine serum (FBS), penicillin-streptomycin mixture, and 0.25% trypsin-EDTA were purchased from Gibco-BRL Life Technologies (Grand Island, NY, USA). Anti-SOD1, anti-HO-1, anti-catalase (CAT), anti-glutathione peroxidase 1 (GPx-1), and anti-Nrf2 were purchased from Santa Cruz Biotechnology (Santa Cruz, CA, USA). Anti-phospho-JNK, anti-ERK1/2, anti-phospho-ERK1/2, anti-phospho-p38, and anti-p38 were purchased from Cell Signaling Technology (Beverly, MA, USA).

### 4.3. HPLC Analysis

The phytochemical characteristics of NNF and the standard compounds galic acid, catechin, epigallocatechin, epicatechin gallate, epicatechin, caffeic acid, quercetin, and apigenin were identified by high performance liquid chromatography-diode array detection (HPLC-DAD) with a Shimadzu Prominence Auto Sampler (SIL-20A) HPLC system (Shimadzu, Kyoto, Japan), equipped with SPD-M20A diode array detector and LC solution 1.22 SP1 software. Reverse-phase chromatographic analysis was carried out using a Phenomenex C18 column (4.6 mm × 250 mm) packed with 5 μm diameter particles. A stepwise gradient of solvent A (acetonitrile) to solvent B (1% formic acid solution) was used with the ration changing each minute as follows: 10% A up to 10 min, which was then changed to obtain 30%, 50%, 60%, 90%, 20%, and 10% A in 15, 20, 25, 30, 35, and 40 min, respectively, at λ = 280 nm. This is in accordance with a slightly varied protocol detailed by Brito et al. [37]. Polyphenolic compounds were identified by comparing retention times with those of available pure standards.

### 4.4. Radical-Scavenging Activity Assays

To evaluate the free radical scavenging activity of NNF, DPPH- [38] and ABTS-radical-scavenging assay [39] was performed in which ascorbic acid was used as a positive control. The following equation was used to calculate the percent inhibition:

(1) $$\mathrm{Radical}-\mathrm{scavenging}\;\mathrm{activity}\;\left(\%\;\mathrm{inhibition}\right)=\left\{\frac{\left({\mathrm{Abs}}_{c}-{\mathrm{Abs}}_{s}\right)}{{\mathrm{Abs}}_{c}}\right\}\times 100$$

where Abs_c_ and Abs_s_ denotes the absorbance of the control and the experimental sample, respectively. All samples were analyzed in triplicate.

A non-enzymatic phenazine methosulfate-nicotinamide adenine dinucleotide (PMS/NADH) system was used to generate the superoxide radical (O_2_^•−^), which reduces NBT to a purple color formazan. The method described by Kumar and Chattopadhyay [12] was adopted for the superoxide scavenging activity of NNF and percent inhibition was calculated by using Equation (1).

The Fe^3+^-ascorbate-EDTA-H_2_O_2_ system (Fenton reaction) was carried out to generate the hydroxyl radical (OH^•^). The hydroxyl radical (OH^•^) scavenging activity of NNF was measured as described in previous method [40] and equation 1 was used to determine the percent inhibition.

For the measurement of reducing power, the ferric reducing antioxidant power (FRAP) assay [41] and the cupric-reducing antioxidant capacity (CUPRAC) [42] was performed and the results were expressed as the ascorbic acid-equivalent antioxidant value (μM).

The oxygen radical absorbance capacity (ORAC) assay [43] was carried out using Trolox, a water-soluble analogue of vitamin E, as a positive control. The experiment was conducted at 37 °C and at pH 7.4 with a blank sample in parallel, and the antioxidant potentiality was calculated as a trolox-equivalent antioxidant value (µM).

### 4.5. DNA Protecting Activity

A previously described method [44] was adopted to evaluate the hydroxyl radical (OH^•^) induced DNA damage protecting activity of NNFE. A mixture of 30 μL ascorbic acid (1mM) and 1 μL copper sulfate (II) (100 μM ) was used to generate the hydroxyl radicals followed by 40 μL of bacteriophage λ DNA (0.1 μg/mL) was exposed to this solution in the presence and absence of NNFE. After incubation at 37 °C for 1 h, the samples fragments were separated by electrophoresis using 1% agarose gel.

### 4.6. Cell Culture and Cell Viability Assay

RAW 264.7 cells and BV2 cells (ATCC, Rockville, MD, USA) were cultured at 37 °C in DMEM supplemented with 10% FBS, streptomycin-penicillin (100 µg/mL each; Hyclone, South Logan, UT, USA) in a humidified atmosphere of 5% CO_2_. An adequate (5 × 10^5^ cells/mL) number of cells were cultured in 96-well plates for 24 h, and then treated with predetermined concentrations of NNFE followed by 24-h incubation; MTT reagent was added to each well and further incubated at 37 °C for 1 h. Finally, 100% DMSO was used to dissolve the intracellular insoluble formazan and the absorbance was measured at 570 nm using a microplate reader (Victor3, PerkinElmer, Waltham, MA, USA) and the percentage viability was calculated [17].

### 4.7. Measurement of Intracellular Reactive Oxygen Species (ROS)

A previously described method [45] was conducted to measure the intracellular ROS generation induced by *t*-BHP. In brief, an adequate (5 × 10^5^/mL) number of cells (RAW 264.7 cells and BV2 cells) were first cultured in 96-well plates for 24 h. The cells were pretreated with indicated concentrations of NNFE. After 1 h, cells were stimulated with *t*-BHP (100 μM) and incubated for 24 h followed by washing with phosphate buffer saline (PBS) twice and then 25 μM 2′,7′-Dichlorofluorescin diacetate (DCFH-DA) was added and incubated at 37 °C for 30 min. The fluorescence intensity was measured at an excitation wavelength of 485 nm and emission wavelength of 528 nm using a fluorescence microplate reader (Victor3, PerkinElmer, Waltham, MA, USA).

### 4.8. Preparation of Cytosolic and Nuclear Protein Fractionation

Cell cultures were harvested after pretreatment with NNFE at the indicated times and concentrations and pelleted by centrifugation at 280× *g* for 10 min followed by washing with 1× PBS twice. A commercially available CelLyticTM NuCLERTM extraction kit Sigma Aldrich (St. Louis, MO, USA). was used to extract the cytosolic and nuclear proteins fraction. Briefly, an ice-cold hypotonic lysis buffer (10 mM HEPES (pH 7.9), 10 mM KCl, 1.5 mM MgCl_2_, 1 mM DTT, and 1× protease inhibitor cocktail) was used to resuspend the cell pellets. It was then incubated on ice for 15 min to allow cells to swell. After addition of 0.25% of NP-40 detergent, the sample was vigorously vortexed for 10 s to disrupt cell membranes followed by centrifugation at 10,000× *g* for 30 s. The cytosolic fraction (supernatant) was separated from the nuclei-enriched fraction (pellet) and was stored at −80 °C. To avoid any cytosolic contamination, the nuclear fraction was washed twice with the hypotonic lysis buffer. Then a hypertonic buffer solution (20 mM HEPES pH 7.9, 1.5 mM MgCl_2_, 0.42 mM NaCl, 25% [*v*/*v*] glycerol, 1 mM DTT, and 1× protease inhibitor cocktail) was used to extract the nuclear proteins from the nuclei using with vigorous agitation for 20 min at room temperature and centrifuged at 16,000× *g* for 10 min. The final supernatant (nuclear extract) was collected and stored at −80 °C.

### 4.9. Transfection of Small Interfering RNA (siRNA)

To carry out the siRNA experiment, an adequate (2 × 10^5^ cells/well) number of cells (RAW 264.7 cells) were first cultured in 6-well plates for 24 h and allowed to grow until ~60% confluent. Then cells were transfected with 10–50 nM siRNA using Lipofectamine RNAiMax (Invitrogen, Carlsbad, CA, USA) according to the manufacturer’s instructions. si-Control RNA and si-Nrf2 RNA were purchased from Santa Cruz Biotechnology (Santa Cruz, CA, USA).

### 4.10. Reverse Transcription-Polymerase Chain Reaction (RT-PCR)

To perform the RT-PCR analysis, first total RNA was isolated using TRI-zol (Invitrogen Co., Carlsbad, CA, USA) following the manufacturer’s instructions. Then cDNA was prepared by using total RNA (2 μg) along with reverse transcriptase (MP Biomedicals, Santa Ana, CA, USA) and oligo(dT) primers and a PCR Thermal Cycler Dice TP600 (Takara Bio Inc., Otsu, Shiga, Japan) was used for amplification of cDNA. After electrophoresis, ethidium bromide staining was performed to detect the PCR products and the Image Lab™ Software, version 5.2.1 (Bio-Rad Laboratories, Hercules, CA, USA) was used to analyze the band intensity. Table S1 showed the specific primers for mouse used in this study.

### 4.11. Preparation of Cell Lysates and Western Blotting

A standard protocol was used to prepare the cell lysates and mixed with 5x SDS-page loading buffer and denatured at 100 °C for 5 min. A nuclear/cytosolic fractionation kit (Cell Biolabs, Inc., San Diego, CA, USA) was used to prepare nuclear protein extract. A 10% SDS-PAGE gel electrophoresis was carried out to separate the sample protein, followed by an electrotransfer to nitrocellulose membranes (Whatman, Dassel, Germany). Then the membranes was blocked using 5% not fat skim milk for 1 h, followed by washing with TBST and incubated overnight with specific primary antibody at 4 °C. The next day, the membrane was washed with TBST for several time, followed by 2 h incubation was performed using anti-goat IgG-horse radish peroxidase (HRP) (Santa Cruz) or anti-rabbit IgG-HRP (Santa Cruz, CA, USA) as secondary antibodies. The antigen-antibody reaction was detected using an enhanced chemiluminescence solution system (Thermo Fisher Scientific, Waltham, MA, USA.). The Image Lab™ Software, version 5.2.1 was used to analyze the band intensity.

### 4.12. Statistical Analysis

Statistical values are expressed as the mean ± SD (*n* = 3) and analyzed using one-way ANOVA. Differences were considered significant if *p* < 0.001, *p* < 0.01, and *p* < 0.05. All analyses were performed using SPSS for Windows, version 10.07 (SPSS, Chicago, IL, USA).

## 5. Conclusions

The involvement of oxidative stress in the etiology and progression of several acute and chronic clinical disorders suggests that antioxidants may have health benefits as prophylactic agents. In the present study, our results showed that NNF has strong antioxidant activity due to the presence of various polyphenolic compounds as confirmed by HPLC. Furthermore, pretreatment of RAW 264.7 cells with NNFE intensely protects the cells against *t*-BHP-induced oxidative stress by attenuating cell death and ROS generation, and futhermore augments the expression of primary antioxidant enzymes and/or Nrf2-mediated HO-1 expression. Moreover, pretreatment with NNFE activated ERK1/2 and p38 signaling pathways, which are pro-survival signaling cascades and might be involved in the cytoprotective effect of NNFE. Finally, present findings give up-to-date insights into the protective effects and mechanisms of *N. nouchali* flower against oxidative stress.

## Supplementary Materials

Supplementary materials can be found at www.mdpi.com/1422-0067/18/10/2069/s1.
