# Supplementary material for: DNA Protecting Activities of Nymphaea nouchali (Burm. f) Flower Extract Attenuate t-BHP-Induced Oxidative Stress Cell Death through Nrf2-Mediated Induction of Heme Oxygenase-1 Expression by Activating MAP-Kinases
Source: Int J Mol Sci. 2017 Sep 28;18(10):2069. doi: 10.3390/ijms18102069 (PMC5666751; doi:10.3390/ijms18102069)
Supplement: Supplementary file 1 [file ijms-18-02069-s001.pptx]

## Slide 1
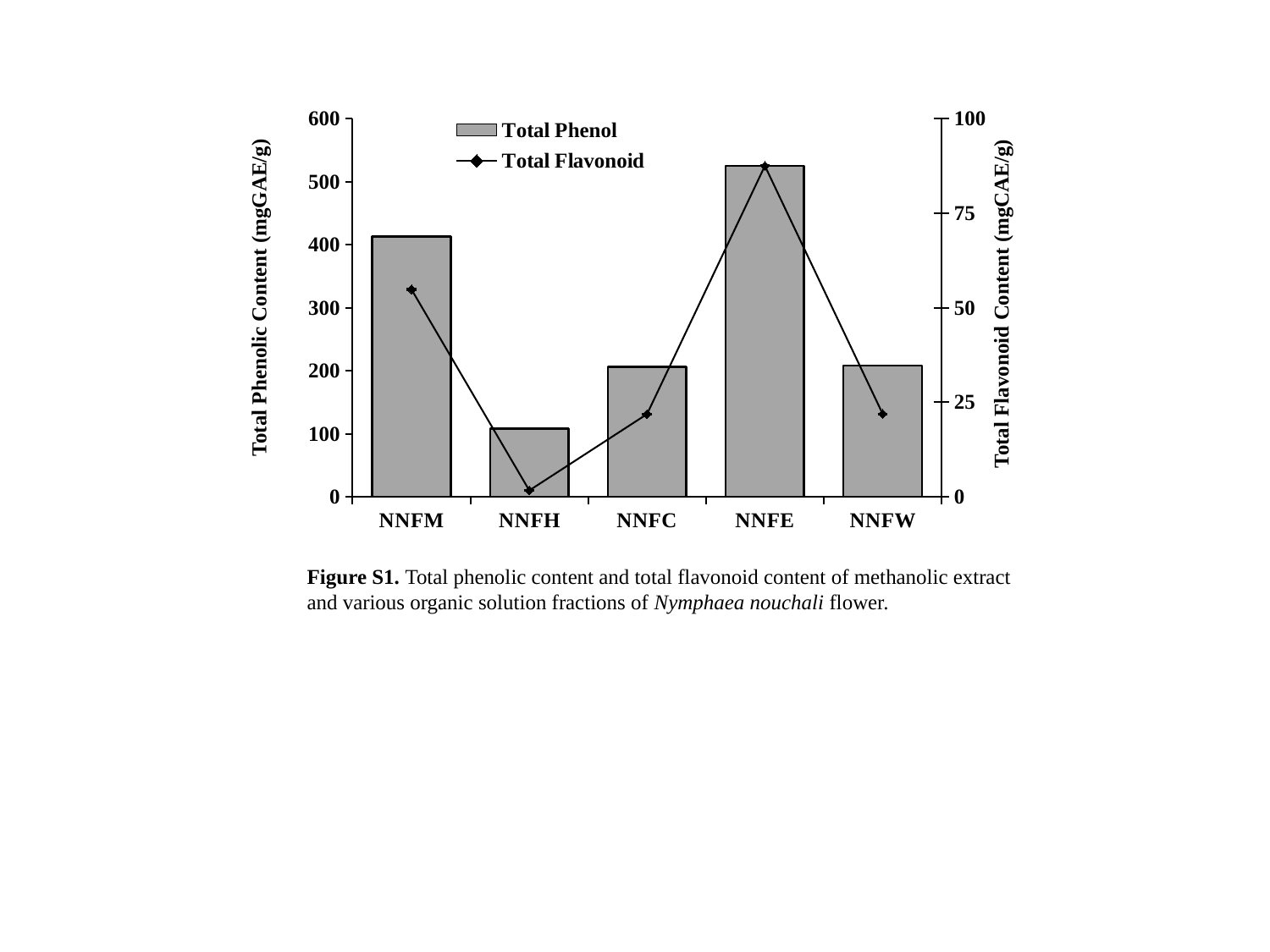

### Chart
| Category | Total Phenol | Total Flavonoid |
|---|---|---|
| NNFM | 413.2271328345996 | 54.8552936829651 |
| NNFH | 108.12699178715759 | 1.6962407946437903 |
| NNFC | 206.50045996056647 | 21.900623376116354 |
| NNFE | 525.1803982118447 | 87.54040751290592 |
| NNFW | 208.28887801433763 | 21.93099436971434 |Total Phenolic Content (mgGAE/g)
Total Flavonoid Content (mgCAE/g)
Figure S1. Total phenolic content and total flavonoid content of methanolic extract
and various organic solution fractions of Nymphaea nouchali flower.

## Slide 2
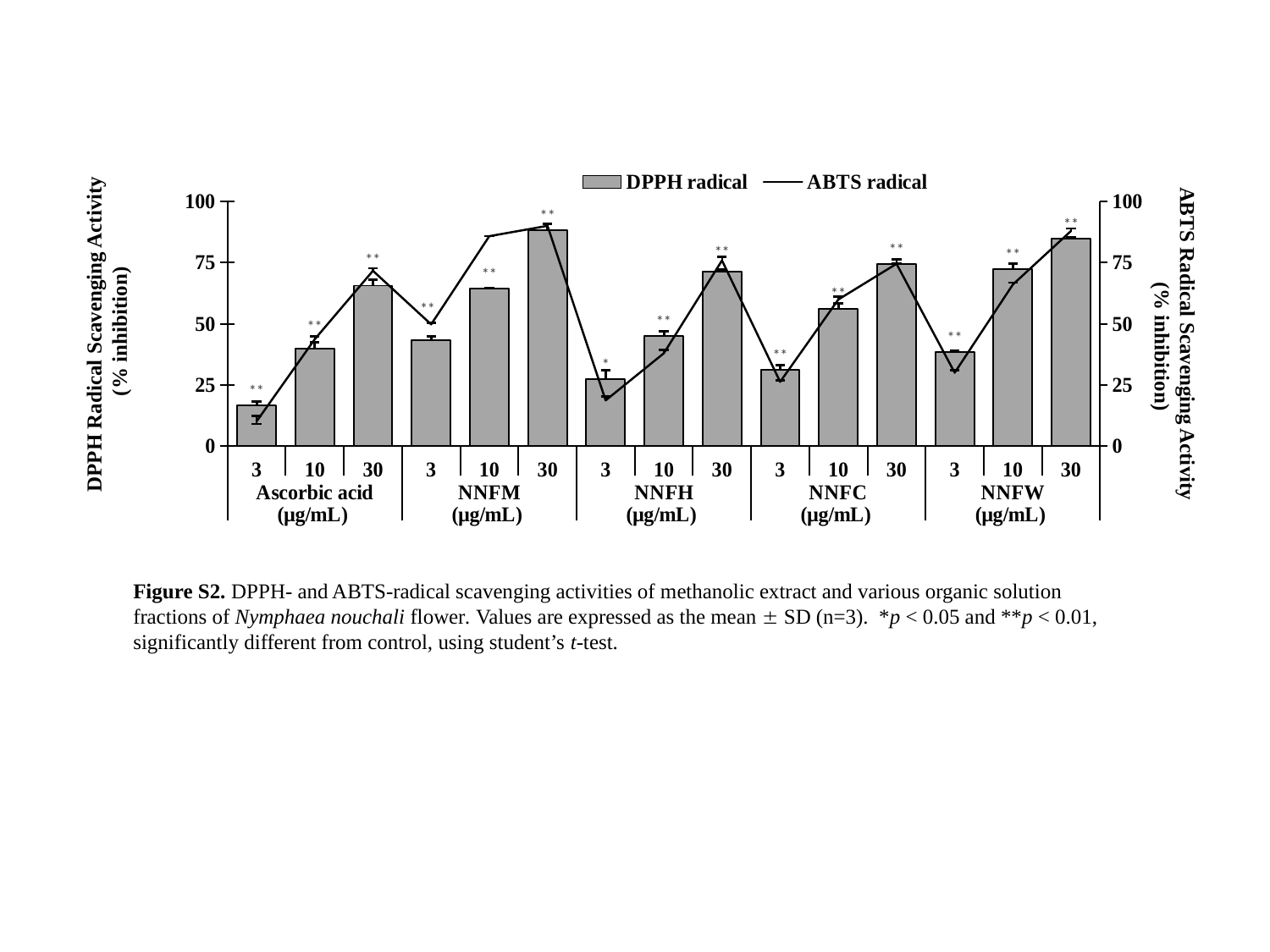

### Chart
| Category | | |
|---|---|---|
| 3 | 16.68121404154979 | 9.969472755671477 |
| 10 | 39.84021320933063 | 43.6782301590319 |
| 30 | 65.60903329742952 | 71.68341704481979 |
| 3 | 43.23851147450796 | 49.72476505623845 |
| 10 | 64.32482480699925 | 85.86186952822736 |
| 30 | 88.48514481679537 | 90.09648586375859 |
| 3 | 27.31702128116467 | 18.586491841718097 |
| 10 | 44.898102310630975 | 37.895857136265086 |
| 30 | 71.21478431799547 | 75.91570944789744 |
| 3 | 31.175876559686596 | 26.171781303568014 |
| 10 | 56.25148449978999 | 59.936791967213104 |
| 30 | 74.49522331912175 | 74.54880241698393 |
| 3 | 38.43154567033426 | 29.912861502691083 |
| 10 | 72.35920266159646 | 66.05003807514457 |
| 30 | 85.00001362184524 | 88.01138051143108 |DPPH Radical Scavenging Activity
(% inhibition)
Figure S2. DPPH- and ABTS-radical scavenging activities of methanolic extract and various organic solution fractions of Nymphaea nouchali flower. Values are expressed as the mean ± SD (n=3). *p < 0.05 and **p < 0.01, significantly different from control, using student’s t-test.
ABTS Radical Scavenging Activity
(% inhibition)

## Slide 3
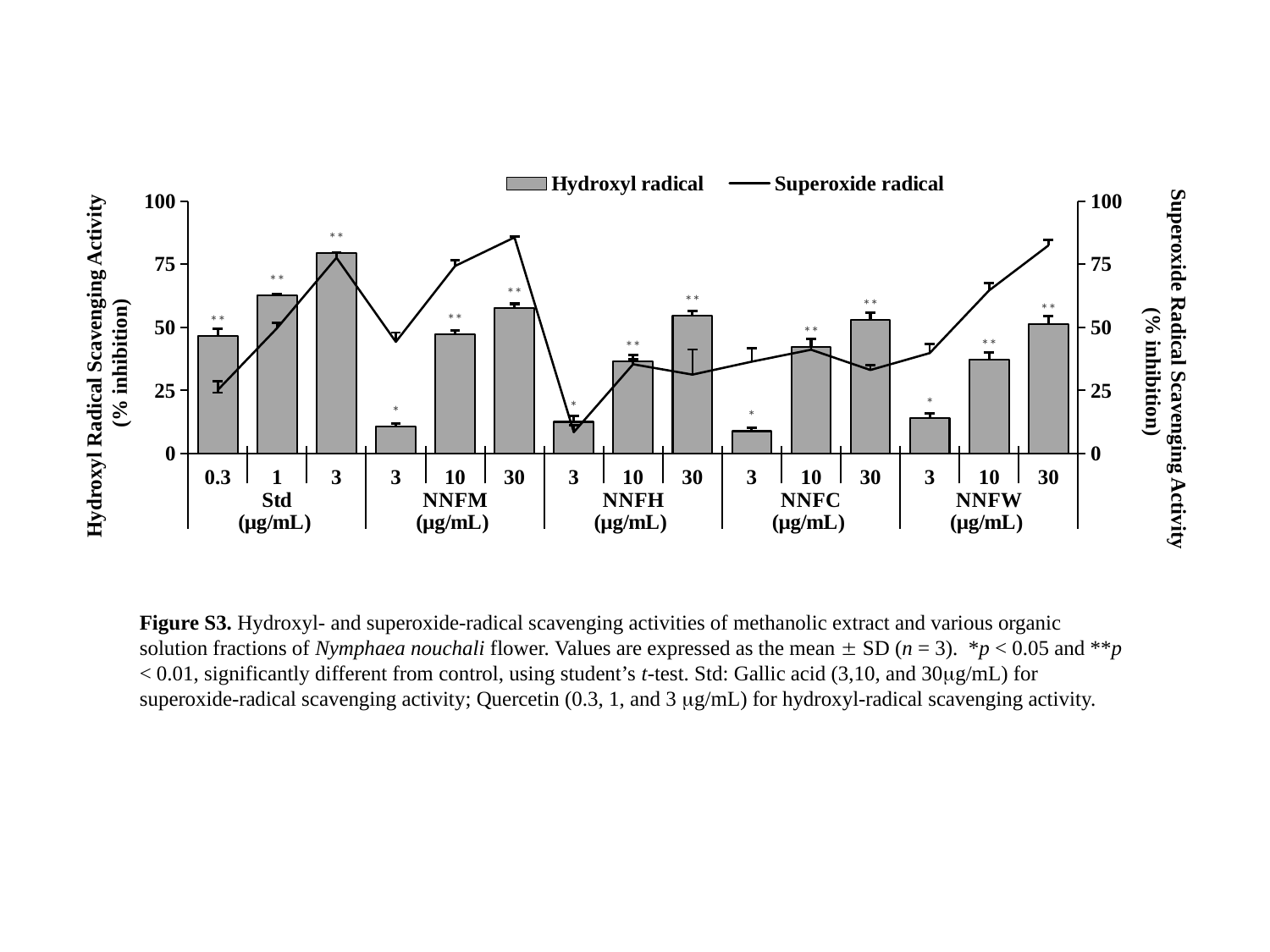

### Chart
| Category | | |
|---|---|---|
| 0.3 | 46.55461282773831 | 25.18164778708074 |
| 1 | 62.758995621529664 | 49.742535221924804 |
| 3 | 79.52312649534277 | 77.59969857191352 |
| 3 | 10.561558331187104 | 44.27787299665988 |
| 10 | 47.25393255701723 | 74.34353059494465 |
| 30 | 57.75542758133715 | 85.66171596800125 |
| 3 | 12.542926337530334 | 8.457021736111967 |
| 10 | 36.51005455861138 | 35.367761000150914 |
| 30 | 54.554205419427284 | 31.27152205153165 |
| 3 | 8.848940632374175 | 36.404892696512455 |
| 10 | 42.35749204045481 | 41.13990003269703 |
| 30 | 53.074559888227974 | 33.081246993431904 |
| 3 | 13.905778492581561 | 39.821801650744334 |
| 10 | 37.32127654867868 | 64.64584934607866 |
| 30 | 51.20783640447081 | 82.47058556909009 |Hydroxyl Radical Scavenging Activity
(% inhibition)
Superoxide Radical Scavenging Activity
(% inhibition)
Figure S3. Hydroxyl- and superoxide-radical scavenging activities of methanolic extract and various organic solution fractions of Nymphaea nouchali flower. Values are expressed as the mean ± SD (n = 3). *p < 0.05 and **p < 0.01, significantly different from control, using student’s t-test. Std: Gallic acid (3,10, and 30μg/mL) for superoxide-radical scavenging activity; Quercetin (0.3, 1, and 3 μg/mL) for hydroxyl-radical scavenging activity.

## Slide 4
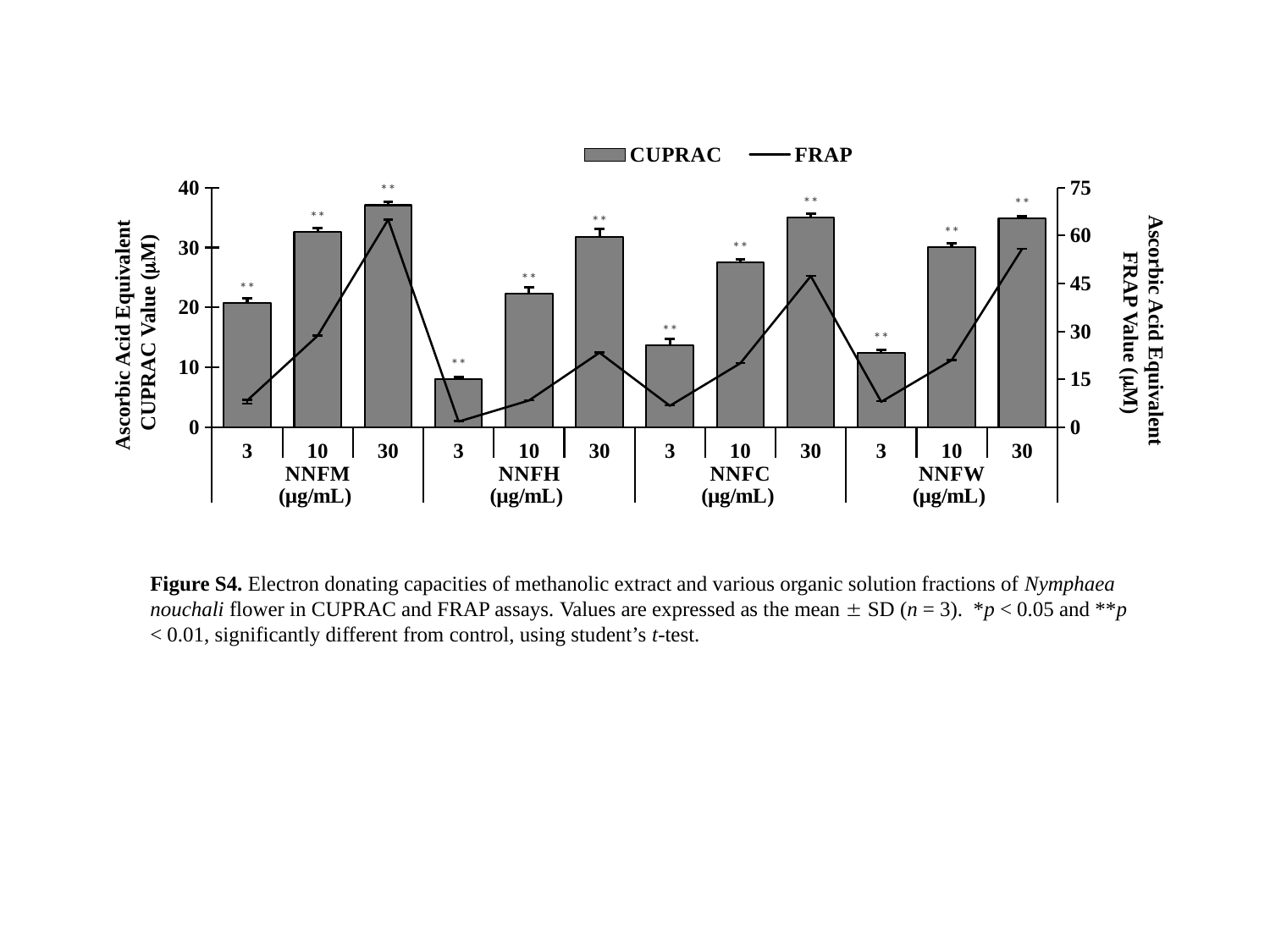

### Chart
| Category | | |
|---|---|---|
| 3 | 20.726764896114418 | 8.40855123297051 |
| 10 | 32.60509361262371 | 28.678605451546854 |
| 30 | 37.05347760289021 | 64.9502872969371 |
| 3 | 8.058616757803849 | 1.812275047949014 |
| 10 | 22.252077496769544 | 8.415224128360812 |
| 30 | 31.81153293088354 | 23.35549036681147 |
| 3 | 13.747919043172516 | 6.7469229072071935 |
| 10 | 27.55492124370884 | 20.03215092471578 |
| 30 | 34.99858572882735 | 47.30373822020982 |
| 3 | 12.478319386755604 | 7.990581828106983 |
| 10 | 30.01972209209448 | 21.004148832431493 |
| 30 | 34.89111271295861 | 55.82955183711837 |Ascorbic Acid Equivalent
 FRAP Value (μM)
Ascorbic Acid Equivalent
CUPRAC Value (μM)
Figure S4. Electron donating capacities of methanolic extract and various organic solution fractions of Nymphaea nouchali flower in CUPRAC and FRAP assays. Values are expressed as the mean ± SD (n = 3). *p < 0.05 and **p < 0.01, significantly different from control, using student’s t-test.

## Slide 5
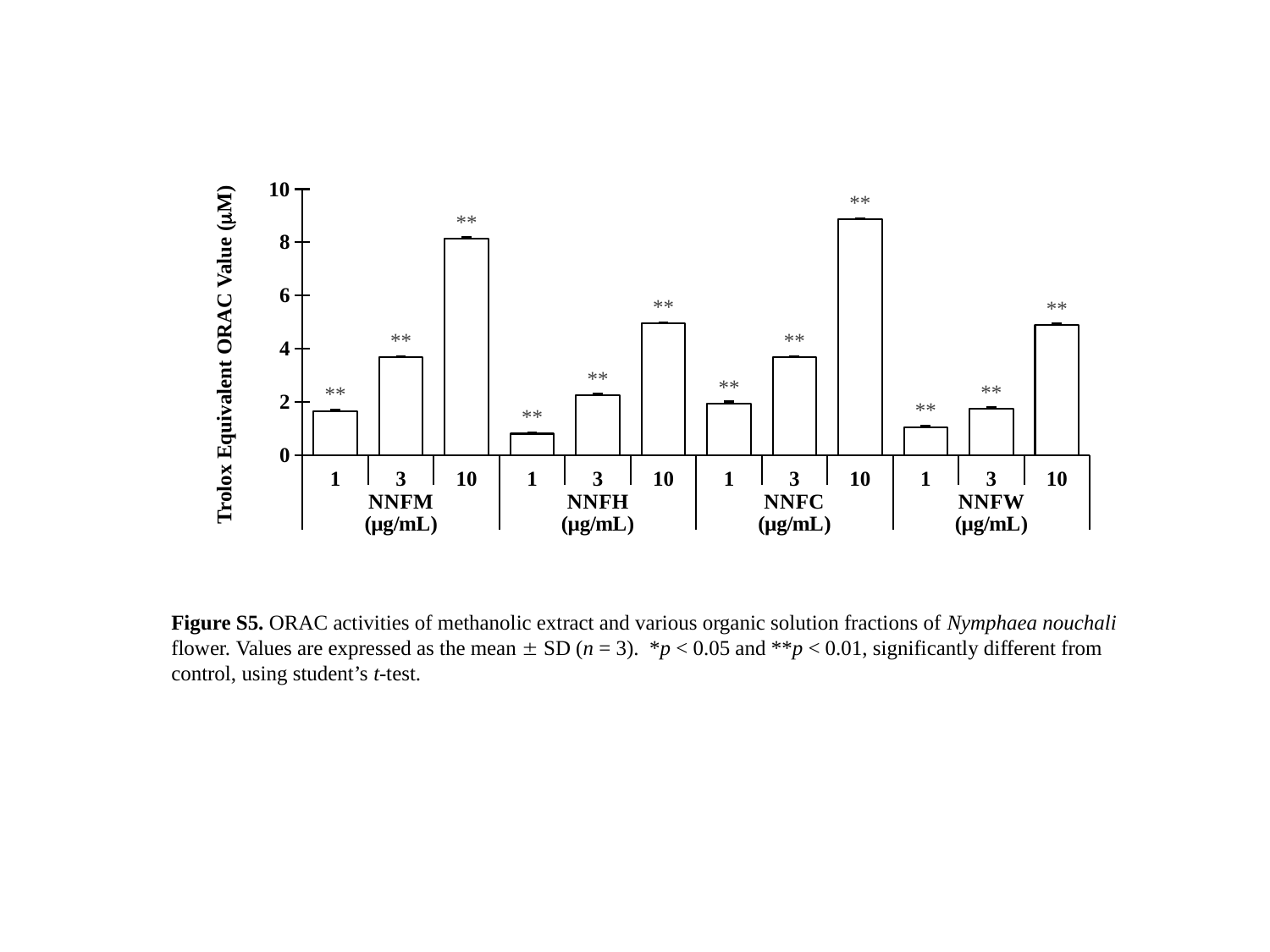

### Chart
| Category | |
|---|---|
| 1 | 1.6574667652032824 |
| 3 | 3.6755191903841906 |
| 10 | 8.14081259905217 |
| 1 | 0.8013571911849003 |
| 3 | 2.2369372146645055 |
| 10 | 4.950215762950832 |
| 1 | 1.9423030689920693 |
| 3 | 3.6801548620221807 |
| 10 | 8.87778278256083 |
| 1 | 1.0534940492470086 |
| 3 | 1.7393512254457963 |
| 10 | 4.900396780517658 |Trolox Equivalent ORAC Value (μM)
Figure S5. ORAC activities of methanolic extract and various organic solution fractions of Nymphaea nouchali flower. Values are expressed as the mean ± SD (n = 3). *p < 0.05 and **p < 0.01, significantly different from control, using student’s t-test.

## Slide 6
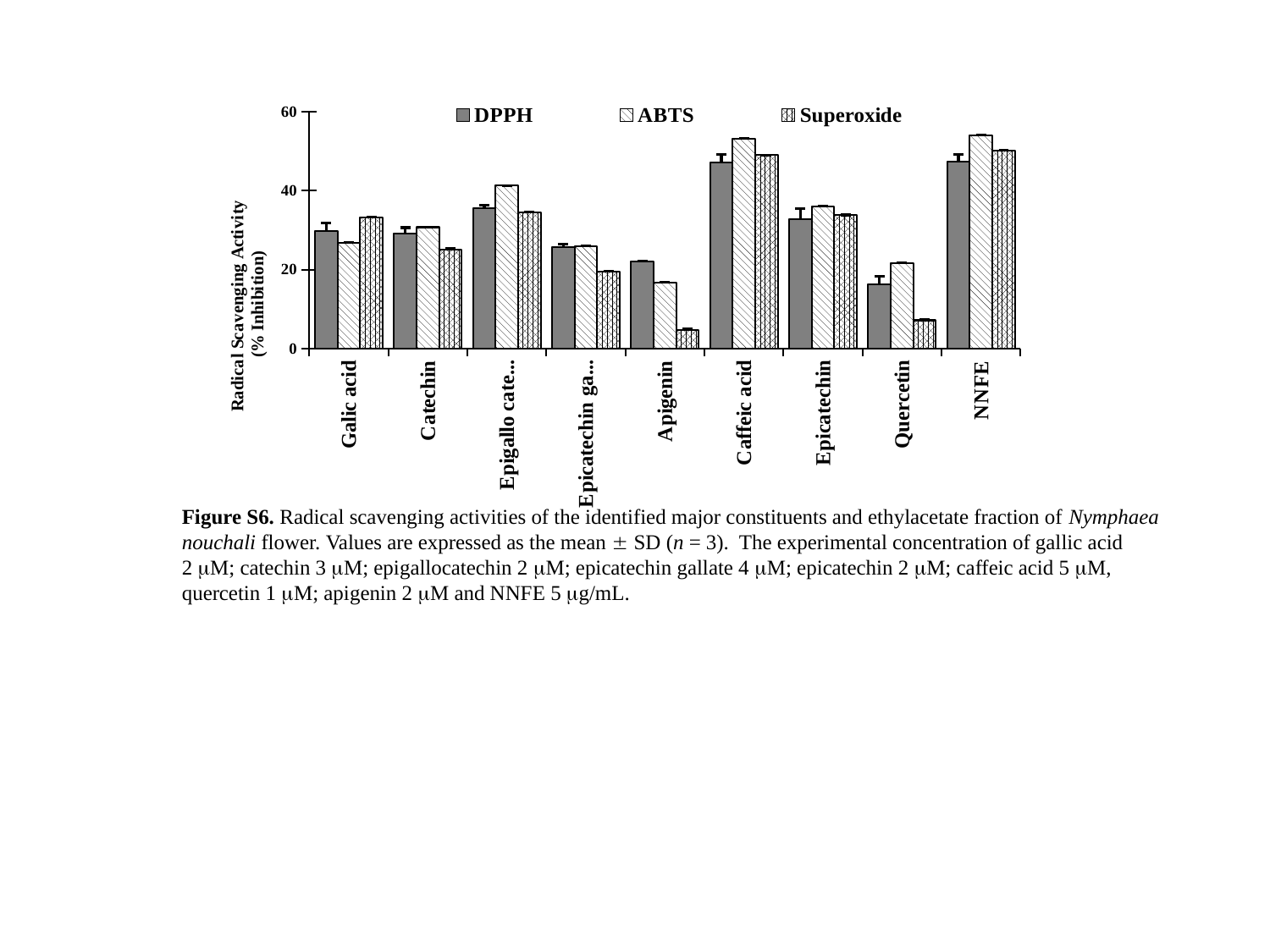

### Chart
| Category | | | |
|---|---|---|---|
| Galic acid | 29.81534657144444 | 26.767946396624723 | 33.287245486905576 |
| Catechin | 29.205848080161065 | 30.77146038840011 | 25.18164778708074 |
| Epigallo catechin | 35.52142449878561 | 41.30458538680956 | 34.54487555329347 |
| Epicatechin gallate | 25.84718386896452 | 25.99995827595175 | 19.60921395469566 |
| Apigenin | 22.151656790708827 | 16.84489865343847 | 4.853149277055034 |
| Caffeic acid | 47.15809111097682 | 53.19897618784932 | 48.99969155969 |
| Epicatechin | 32.80062420430862 | 36.0863177254636 | 33.87813412147586 |
| Quercetin | 16.328803594353513 | 21.720665638235758 | 7.248416466592999 |
| NNFE | 47.41855502558701 | 54.07231776057525 | 50.2147768913264 |Figure S6. Radical scavenging activities of the identified major constituents and ethylacetate fraction of Nymphaea nouchali flower. Values are expressed as the mean ± SD (n = 3). The experimental concentration of gallic acid
2 μM; catechin 3 μM; epigallocatechin 2 μM; epicatechin gallate 4 μM; epicatechin 2 μM; caffeic acid 5 μM, quercetin 1 μM; apigenin 2 μM and NNFE 5 μg/mL.

## Slide 7
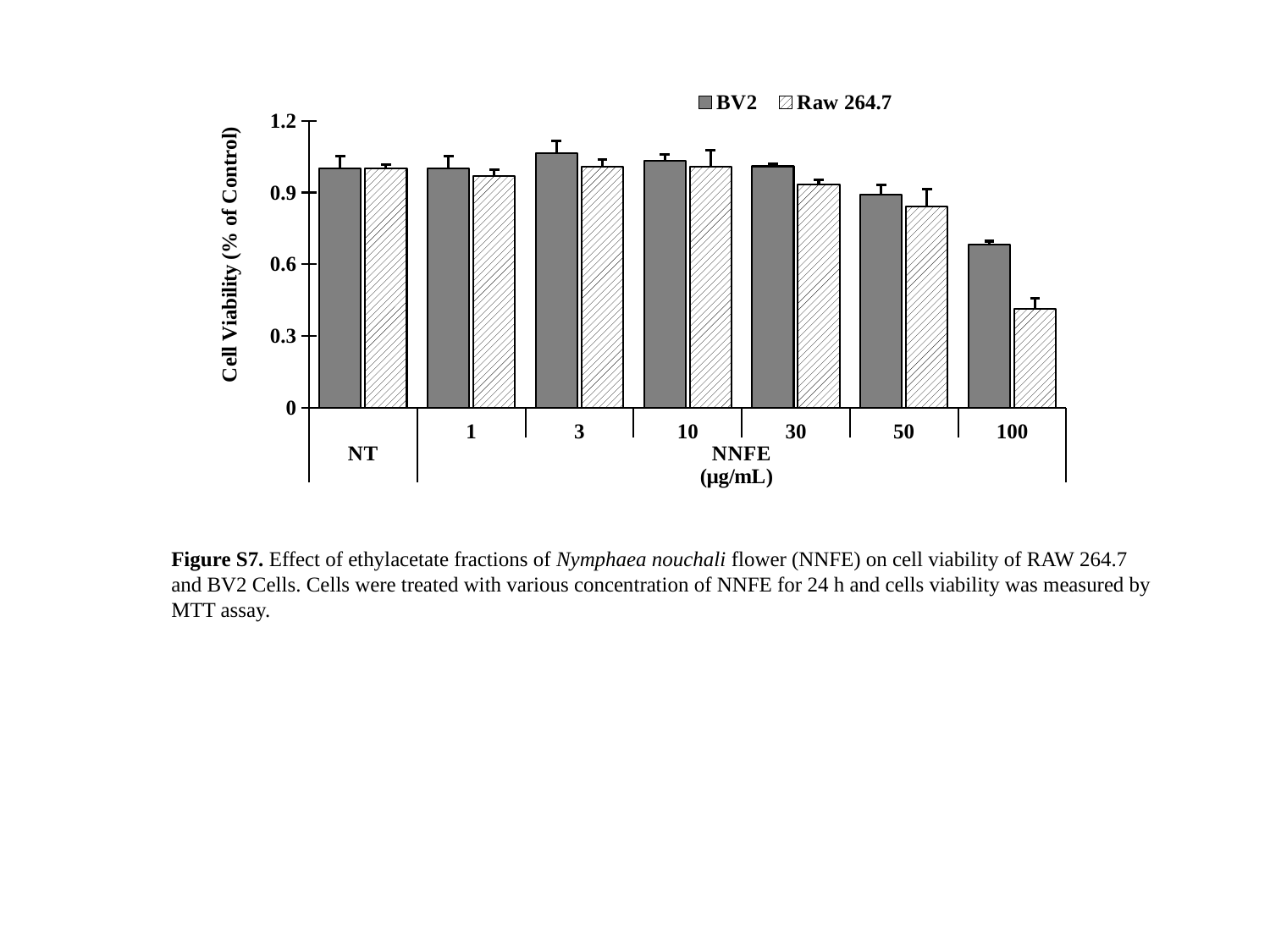

### Chart
| Category | | |
|---|---|---|
| | 1.0 | 1.0 |
| 1 | 1.0 | 0.9676390064657185 |
| 3 | 1.0662041627770127 | 1.009349728724997 |
| 10 | 1.0325009599772879 | 1.009822562962451 |
| 30 | 1.009919421262543 | 0.9323164476731043 |
| 50 | 0.8903690709710674 | 0.8416377495673926 |
| 100 | 0.6826406305658272 | 0.41408525296853665 |Figure S7. Effect of ethylacetate fractions of Nymphaea nouchali flower (NNFE) on cell viability of RAW 264.7 and BV2 Cells. Cells were treated with various concentration of NNFE for 24 h and cells viability was measured by MTT assay.

## Slide 8
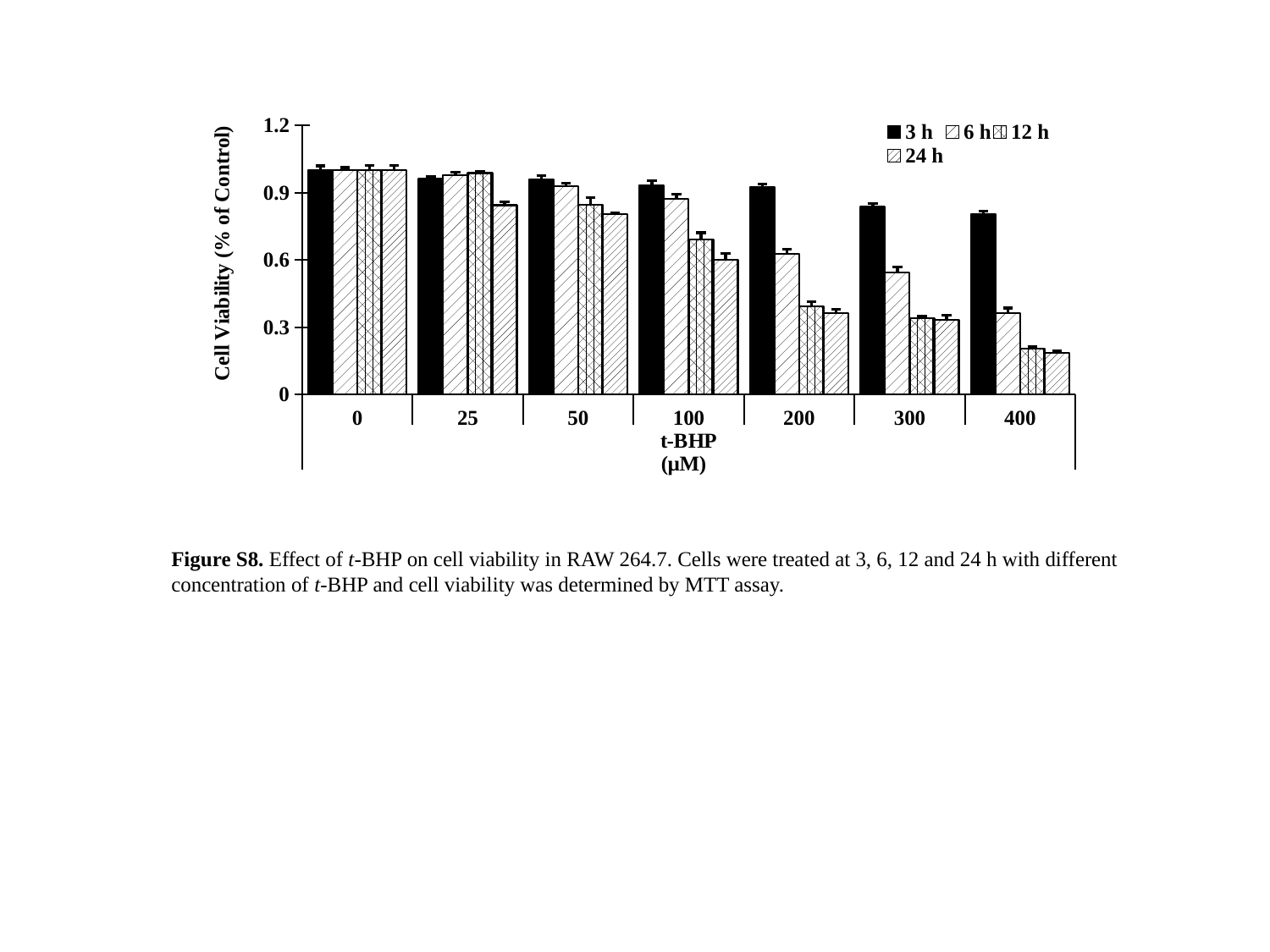

### Chart
| Category | 3 h | 6 h | 12 h | 24 h |
|---|---|---|---|---|
| 0 | 1.0 | 1.0 | 1.0 | 1.0 |
| 25 | 0.9637970932827443 | 0.979865108149209 | 0.9879832756553125 | 0.8446531964762118 |
| 50 | 0.958599712740565 | 0.930034919409166 | 0.8475085327173979 | 0.8062846497576351 |
| 100 | 0.9329668793774447 | 0.8713889517129574 | 0.6920594016571898 | 0.6013069288749088 |
| 200 | 0.9240615127826677 | 0.6267243348403486 | 0.39188857766872726 | 0.3644357419435335 |
| 300 | 0.8372208405232177 | 0.5438231023627929 | 0.339866203066441 | 0.33169938458300047 |
| 400 | 0.8046063379839681 | 0.3642391237033731 | 0.20428490245068062 | 0.18628054426570195 |Figure S8. Effect of t-BHP on cell viability in RAW 264.7. Cells were treated at 3, 6, 12 and 24 h with different concentration of t-BHP and cell viability was determined by MTT assay.

## Slide 9
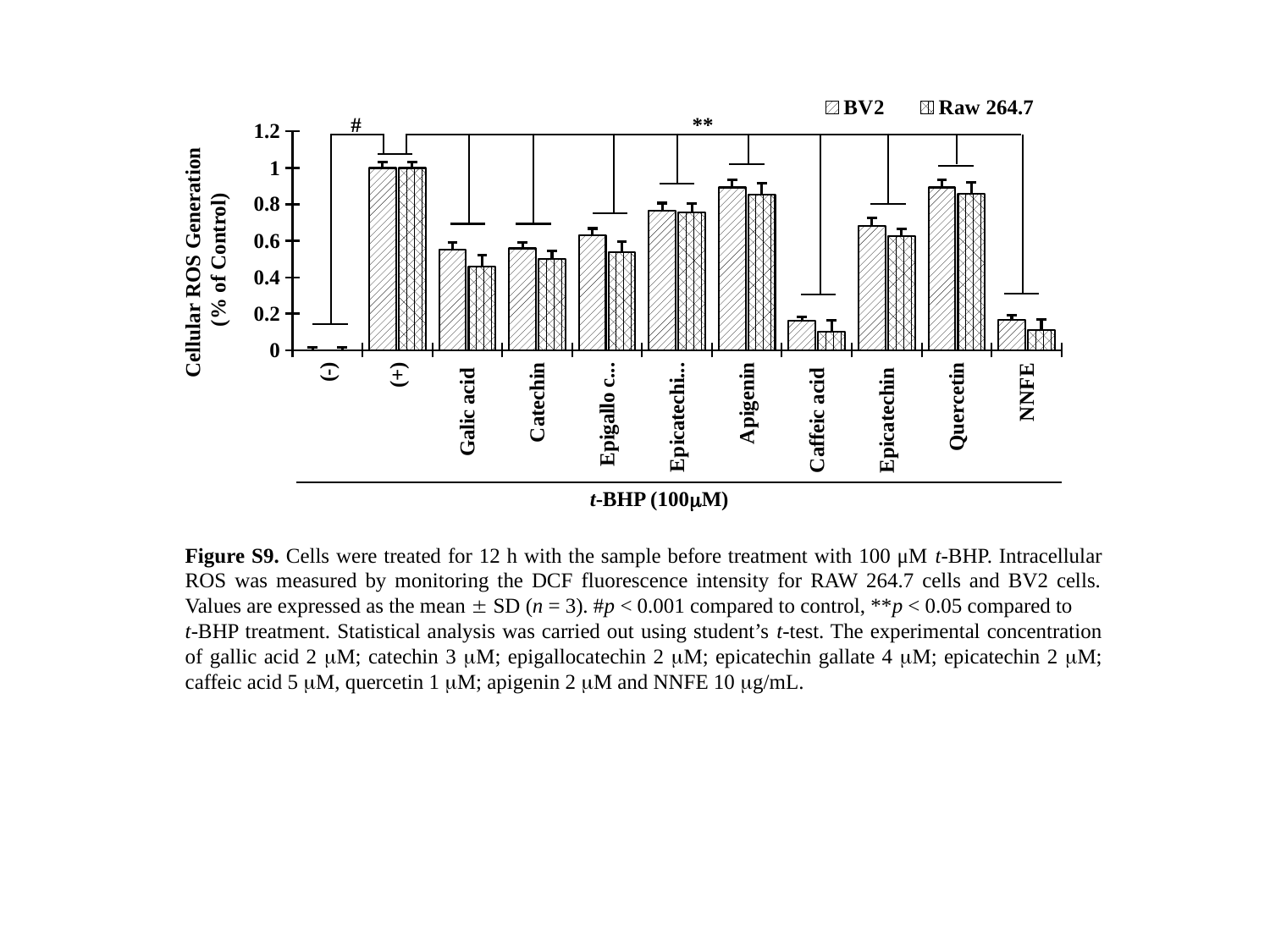

### Chart
| Category | | |
|---|---|---|
| (-) | 0.0 | 0.0 |
| (+) | 1.0 | 1.0 |
| Galic acid | 0.5496597602751064 | 0.46030170254818165 |
| Catechin | 0.5583756838476132 | 0.49873312906966716 |
| Epigallo catechin | 0.6292727638155438 | 0.5363274957244517 |
| Epicatechin gallate | 0.7637784388948888 | 0.7548147864030778 |
| Apigenin | 0.892983397714949 | 0.8555834826202894 |
| Caffeic acid | 0.1586667815155189 | 0.10223975560559363 |
| Epicatechin | 0.6833621559779919 | 0.6261745536808825 |
| Quercetin | 0.892983397714949 | 0.8589678217039909 |
| NNFE | 0.16564915000806146 | 0.10811120787813974 |#
**
Cellular ROS Generation
(% of Control)
t-BHP (100μM)
Figure S9. Cells were treated for 12 h with the sample before treatment with 100 μM t-BHP. Intracellular ROS was measured by monitoring the DCF fluorescence intensity for RAW 264.7 cells and BV2 cells. Values are expressed as the mean ± SD (n = 3). #p < 0.001 compared to control, **p < 0.05 compared to
t-BHP treatment. Statistical analysis was carried out using student’s t-test. The experimental concentration of gallic acid 2 μM; catechin 3 μM; epigallocatechin 2 μM; epicatechin gallate 4 μM; epicatechin 2 μM; caffeic acid 5 μM, quercetin 1 μM; apigenin 2 μM and NNFE 10 μg/mL.

## Slide 10
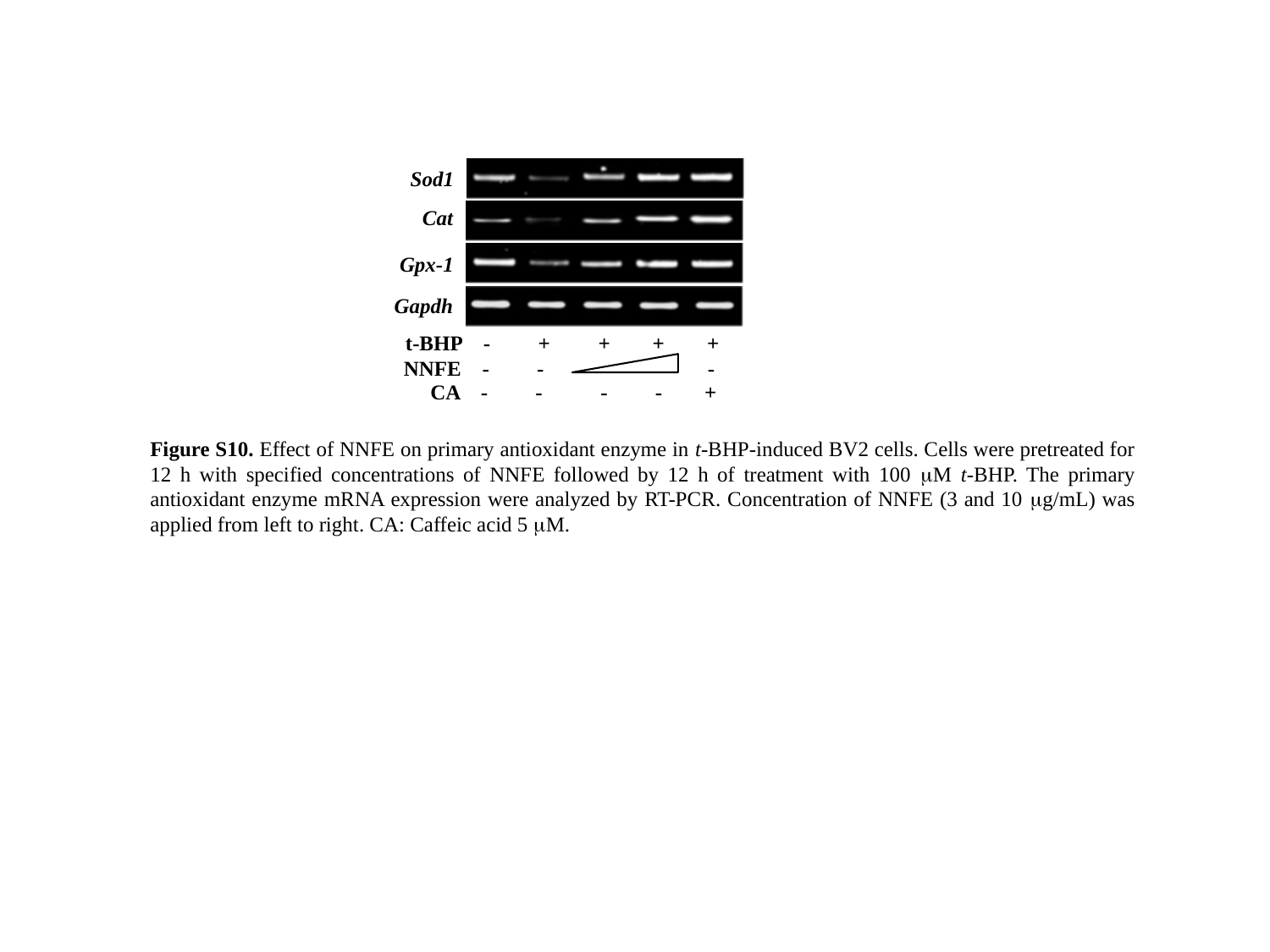

Sod1
Cat
Gpx-1
Gapdh
t-BHP - + + + +
NNFE - - -
CA - - - - +
Figure S10. Effect of NNFE on primary antioxidant enzyme in t-BHP-induced BV2 cells. Cells were pretreated for 12 h with specified concentrations of NNFE followed by 12 h of treatment with 100 μM t-BHP. The primary antioxidant enzyme mRNA expression were analyzed by RT-PCR. Concentration of NNFE (3 and 10 μg/mL) was applied from left to right. CA: Caffeic acid 5 μM.

## Slide 11
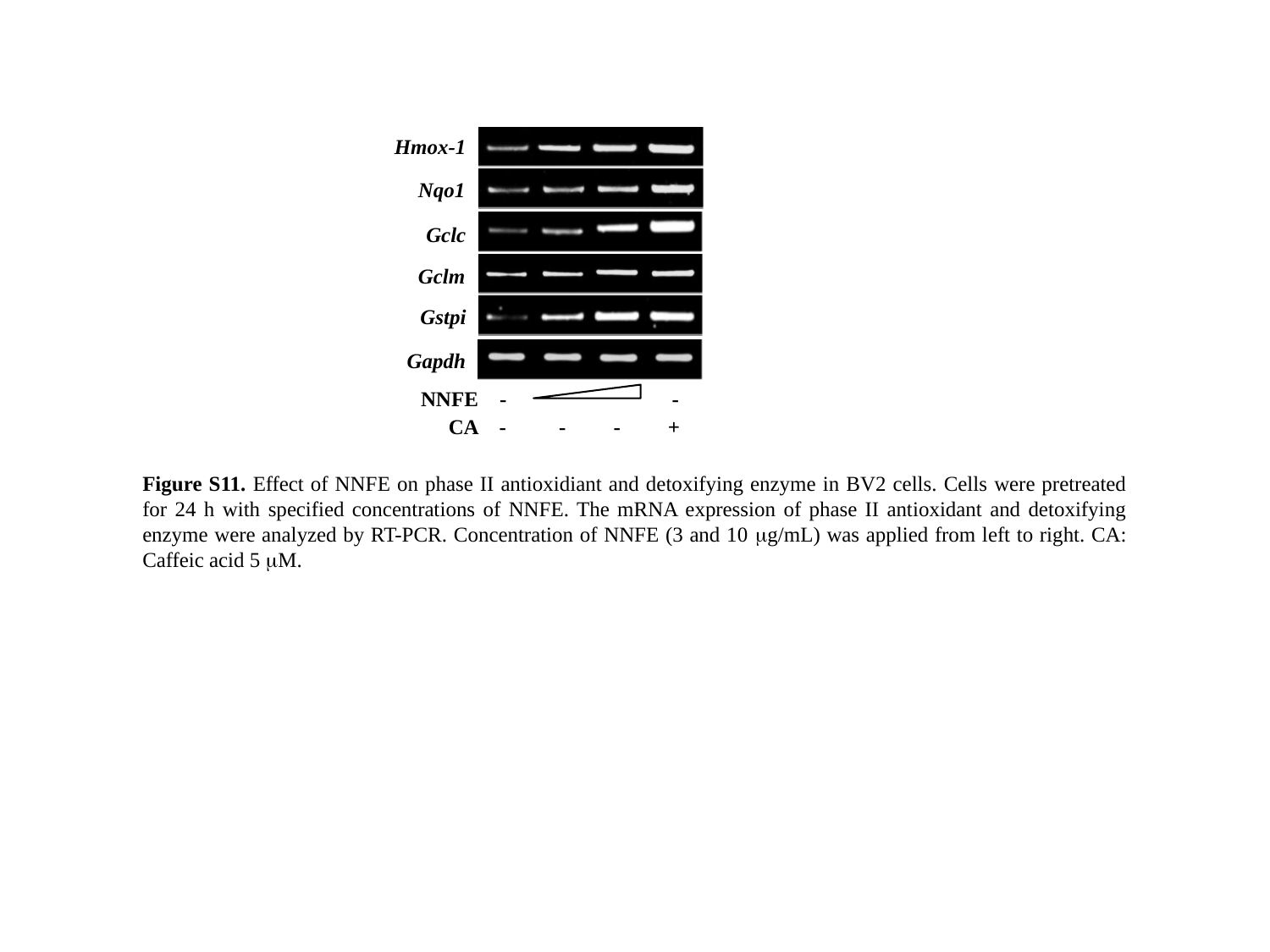

Hmox-1
Nqo1
Gclc
Gclm
Gstpi
Gapdh
NNFE - -
CA - - - +
Figure S11. Effect of NNFE on phase II antioxidiant and detoxifying enzyme in BV2 cells. Cells were pretreated for 24 h with specified concentrations of NNFE. The mRNA expression of phase II antioxidant and detoxifying enzyme were analyzed by RT-PCR. Concentration of NNFE (3 and 10 μg/mL) was applied from left to right. CA: Caffeic acid 5 μM.

## Slide 12
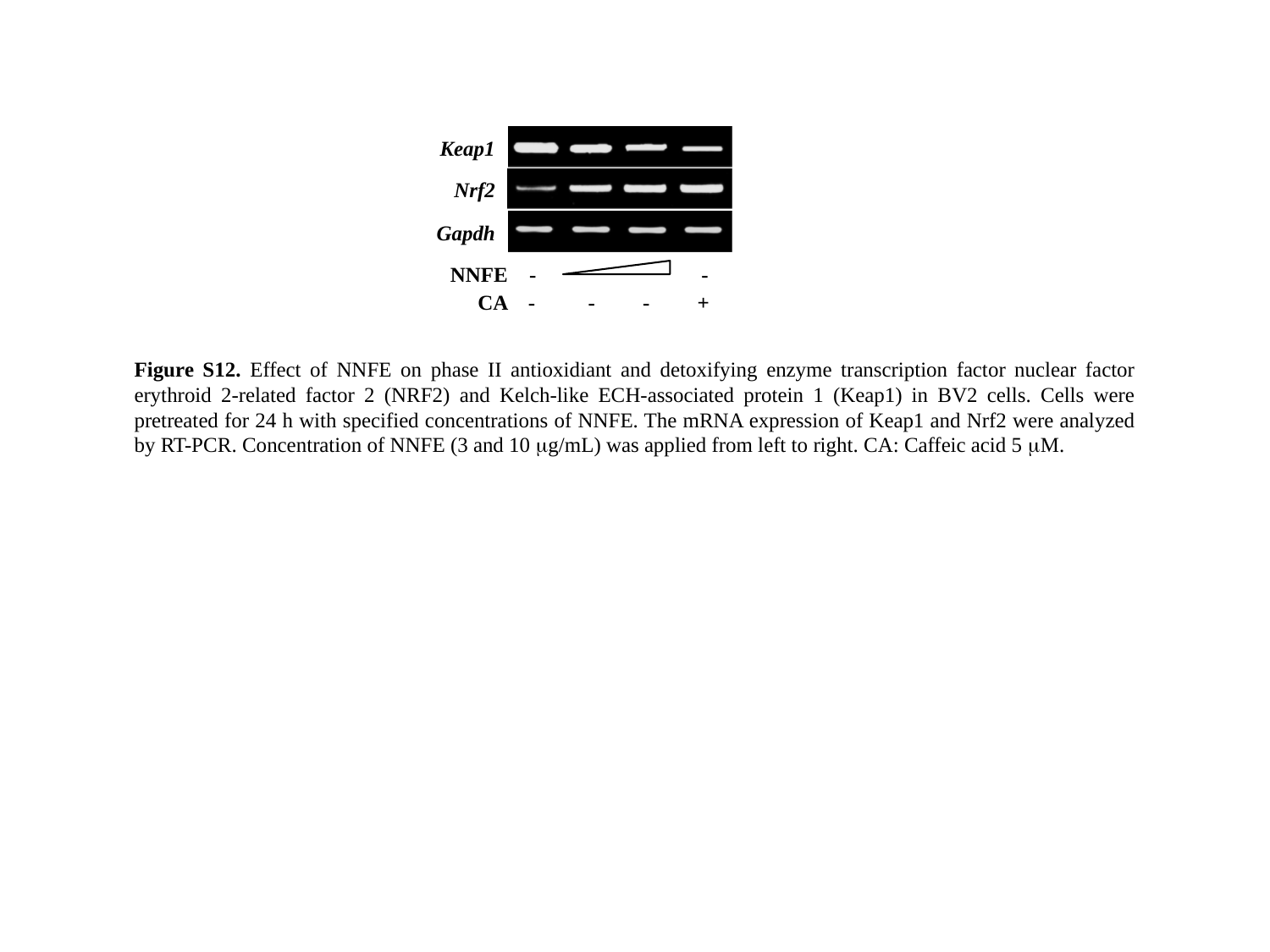

Keap1
Nrf2
Gapdh
NNFE - -
CA - - - +
Figure S12. Effect of NNFE on phase II antioxidiant and detoxifying enzyme transcription factor nuclear factor erythroid 2-related factor 2 (NRF2) and Kelch-like ECH-associated protein 1 (Keap1) in BV2 cells. Cells were pretreated for 24 h with specified concentrations of NNFE. The mRNA expression of Keap1 and Nrf2 were analyzed by RT-PCR. Concentration of NNFE (3 and 10 μg/mL) was applied from left to right. CA: Caffeic acid 5 μM.

## Slide 13
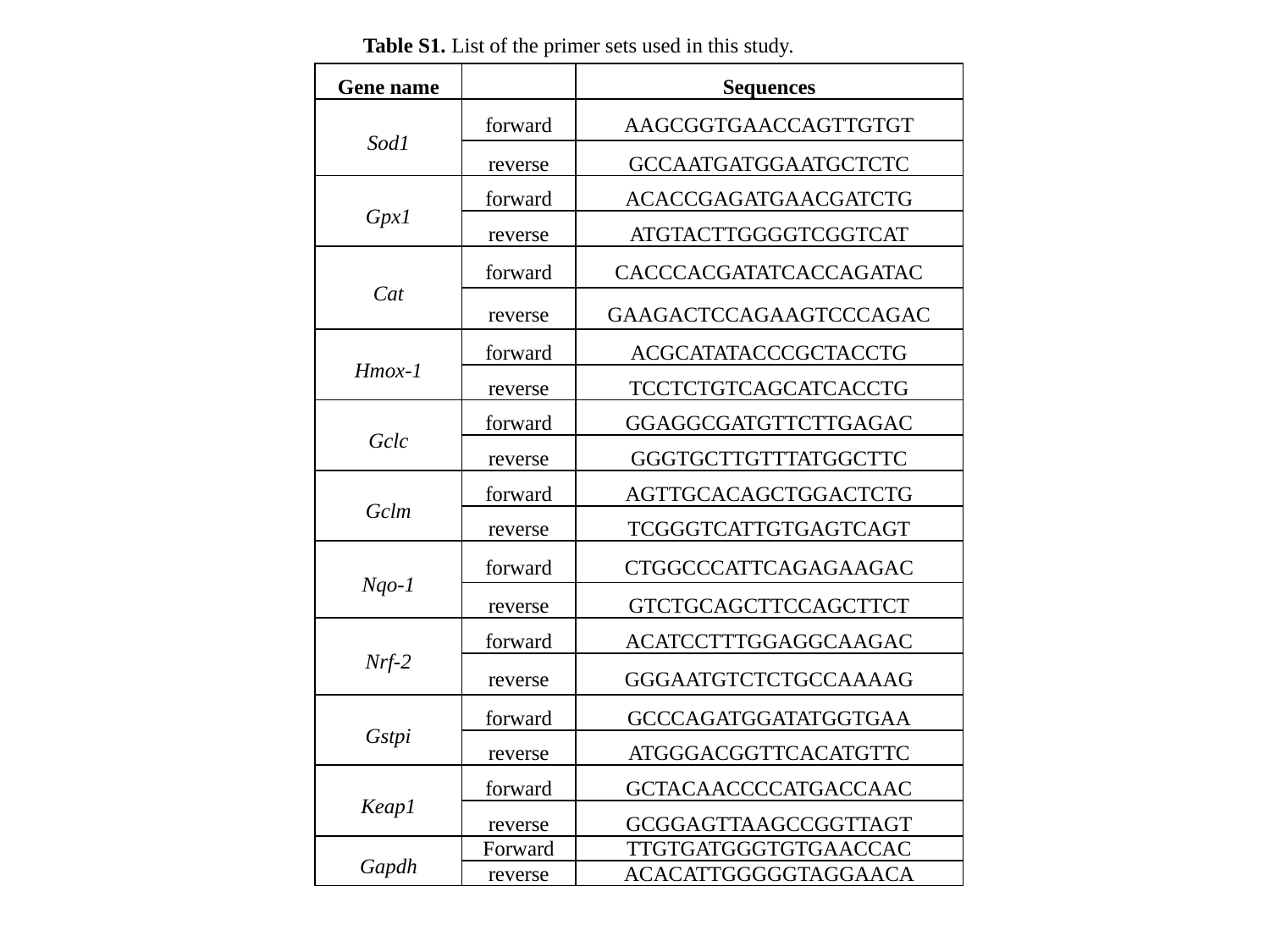

Table S1. List of the primer sets used in this study.
| Gene name | | Sequences |
| --- | --- | --- |
| Sod1 | forward | AAGCGGTGAACCAGTTGTGT |
| | reverse | GCCAATGATGGAATGCTCTC |
| Gpx1 | forward | ACACCGAGATGAACGATCTG |
| | reverse | ATGTACTTGGGGTCGGTCAT |
| Cat | forward | CACCCACGATATCACCAGATAC |
| | reverse | GAAGACTCCAGAAGTCCCAGAC |
| Hmox-1 | forward | ACGCATATACCCGCTACCTG |
| | reverse | TCCTCTGTCAGCATCACCTG |
| Gclc | forward | GGAGGCGATGTTCTTGAGAC |
| | reverse | GGGTGCTTGTTTATGGCTTC |
| Gclm | forward | AGTTGCACAGCTGGACTCTG |
| | reverse | TCGGGTCATTGTGAGTCAGT |
| Nqo-1 | forward | CTGGCCCATTCAGAGAAGAC |
| | reverse | GTCTGCAGCTTCCAGCTTCT |
| Nrf-2 | forward | ACATCCTTTGGAGGCAAGAC |
| | reverse | GGGAATGTCTCTGCCAAAAG |
| Gstpi | forward | GCCCAGATGGATATGGTGAA |
| | reverse | ATGGGACGGTTCACATGTTC |
| Keap1 | forward | GCTACAACCCCATGACCAAC |
| | reverse | GCGGAGTTAAGCCGGTTAGT |
| Gapdh | Forward | TTGTGATGGGTGTGAACCAC |
| | reverse | ACACATTGGGGGTAGGAACA |
